# A comparative analysis of conduction system pacing and biventricular pacing in patients undergoing atrioventricular node ablation: a systematic review and meta-analysis

**DOI:** 10.1093/europace/euaf106

**Published:** 2025-07-11

**Authors:** Akash Mavilakandy, Ahmed M Abdelrazik, Khaled Abouelmagd, Ivelin Koev, Ravi Chotalia, Sachin Sudhakaran, Abdulmalik Idris Koya, Ibrahim Antoun, Hany Eldeeb, Hisham Ahamed, Harshil Dhutia, Riyaz Somani, G Andre Ng, Mokhtar Ibrahim

**Affiliations:** Department of Cardiology, Glenfield Hospital, University Hospitals of Leicester NHS Trust, Leicester LE3 9QP, UK; Department of Cardiovascular Sciences, University of Leicester, Leicester LE3 9QP, UK; Department of Cardiology, Glenfield Hospital, University Hospitals of Leicester NHS Trust, Leicester LE3 9QP, UK; Department of Cardiovascular Sciences, University of Leicester, Leicester LE3 9QP, UK; Cardiology Department, Faculty of Medicine, Al-Azhar University, New Damietta, Egypt; Department of Cardiology, Glenfield Hospital, University Hospitals of Leicester NHS Trust, Leicester LE3 9QP, UK; Department of Cardiovascular Sciences, University of Leicester, Leicester LE3 9QP, UK; Department of Cardiology, Glenfield Hospital, University Hospitals of Leicester NHS Trust, Leicester LE3 9QP, UK; Department of Cardiology, Glenfield Hospital, University Hospitals of Leicester NHS Trust, Leicester LE3 9QP, UK; Department of Cardiovascular Sciences, University of Leicester, Leicester LE3 9QP, UK; Department of Cardiology, Glenfield Hospital, University Hospitals of Leicester NHS Trust, Leicester LE3 9QP, UK; Department of Cardiovascular Sciences, University of Leicester, Leicester LE3 9QP, UK; Department of Cardiology, Glenfield Hospital, University Hospitals of Leicester NHS Trust, Leicester LE3 9QP, UK; Department of Cardiovascular Sciences, University of Leicester, Leicester LE3 9QP, UK; Department of Cardiology, Amrita Institute of Medical Sciences and Research, Kochi, KL 682041, India; Department of Cardiology, Glenfield Hospital, University Hospitals of Leicester NHS Trust, Leicester LE3 9QP, UK; Department of Cardiology, Glenfield Hospital, University Hospitals of Leicester NHS Trust, Leicester LE3 9QP, UK; Department of Cardiovascular Sciences, University of Leicester, Leicester LE3 9QP, UK; Department of Cardiology, Glenfield Hospital, University Hospitals of Leicester NHS Trust, Leicester LE3 9QP, UK; Department of Cardiovascular Sciences, University of Leicester, Leicester LE3 9QP, UK; National Institute for Health Research Leicester Biomedical Research Centre, Leicester LE3 9QP, UK; Department of Cardiology, Glenfield Hospital, University Hospitals of Leicester NHS Trust, Leicester LE3 9QP, UK; Department of Cardiovascular Sciences, University of Leicester, Leicester LE3 9QP, UK; Cardiology Department, Ain Shams University, Cairo, Egypt

**Keywords:** Conduction system pacing, Biventricular pacing, Atrioventricular node ablation, Atrial fibrillation

## Abstract

**Aims:**

Atrioventricular node ablation (AVNA) with permanent pacemaker implantation is an established rate-control treatment approach for patients with AF with uncontrolled ventricular rates. Conduction system pacing (CSP) utilizing His bundle pacing (HBP) or left bundle branch area pacing (LBBAP) has advanced as a treatment alternative to standard right ventricular pacing in addition to biventricular pacing (BVP). This systematic review and meta-analysis aim to provide a comprehensive summary and evaluation of clinical outcomes in the literature for CSP in comparison to BVP in conjunction with AVNA.

**Methods and results:**

This study protocol was registered in the PROSPERO registry (CRD42024510974), and the review was conducted as per the PRISMA guidelines. Databases were searched for relevant studies from inception till 11 January 2024. Results were synthesized using a random effects meta-analysis. From a total of 259 references identified, 122 full texts were assessed, and 25 studies were included in the systematic review. Of these included studies, five were used for comparative meta-analysis. A total of 1652 (HBP 1069 and LBBAP 644) and 369 patients received CSP and BVP implantation with AVNA, respectively. Conduction system pacing resulted in a narrower QRS duration (QRSd) with a change of −35.8 ms (95% CI −61.8 to −9.72; *P* < 0.05; I^2^ = 96.3%) vs. BVP. Conduction system pacing also resulted in better symptomatic improvement in from of NYHA reduction (MD −0.53, 95% CI −1.01 to −0.04, I^2^ = 62.1; *P* = 0.03). For left ventricular ejection fraction, a non-significant weighted mean increases of 3.36% (95% CI −0.75–7.47%; *P* = 0.11, I^2^ = 68.5%) was observed following CSP implantation in comparison to BVP. Conduction system pacing showed no significant differences in procedural and fluoroscopy times and had comparable periprocedural complications. His bundle pacing demonstrated a non-significant reduction in the events of acute threshold elevation in comparison to BVP (Log odds ratio −0.69, 95% CI −2.05–0.66, I^2^ = 0.00; *P* = 0.32).

**Conclusion:**

Conduction system pacing with AVNA is a safe and feasible treatment option for symptomatic (AF) patients undergoing a pace and ablate strategy, offering an alternative to BVP. Overall, CSP results in a narrower QRS duration while providing comparable clinical and echocardiographic outcomes.

## Introduction

Atrial fibrillation (AF) is a highly prevalent and clinically significant arrhythmia that is associated with increased morbidity and mortality.^1,2^ AF and heart failure (HF) are particularly linked, as both share common risk factors and potentiate the risk and severity of one another.^3,4^

Several randomized controlled trials (RCTs) have observed similar efficacy with rate vs. rhythm control strategies for treatment of AF.^5,6^ Catheter ablation remains a key strategy for rhythm control in patients with symptomatic atrial fibrillation who do not respond to antiarrhythmic medications or as a first-line option in a selected group of patients. However, some patients may not be suitable candidates for rhythm control due to comorbidities, while others continue to experience symptoms despite multiple ablation attempts. Therefore, pharmacotherapy constitutes a significant proponent of available approaches for rate-control strategy. Atrioventricular node ablation (AVNA) with subsequent pacing is the last and solid treatment modality for patients with AF exhibiting symptoms and high ventricular rates refractory to pharmacological management. The AHA/ACC/HRS Atrial Fibrillation Practise Guidelines (2014) recommend AVNA with permanent right ventricular pacing (RVP) as a class IIa evidence for rate control in AF when pharmacological strategies are inadequate and rhythm control cannot be attained.^7^ Cardiac resynchronization therapy (CRT) through biventricular pacing (BVP) is considered for patients exhibiting a reduced left ventricular ejection fraction (LVEF), which are referred for AVNA.^7^ CRT has also demonstrated potential benefit in candidates referred for AVNA with a narrow QRS complex as the APAF-CRT trial reported improved outcomes with regard to hospitalization and quality of life (QOL) in comparison to pharmacotherapy.^8^

**Figure 1:**
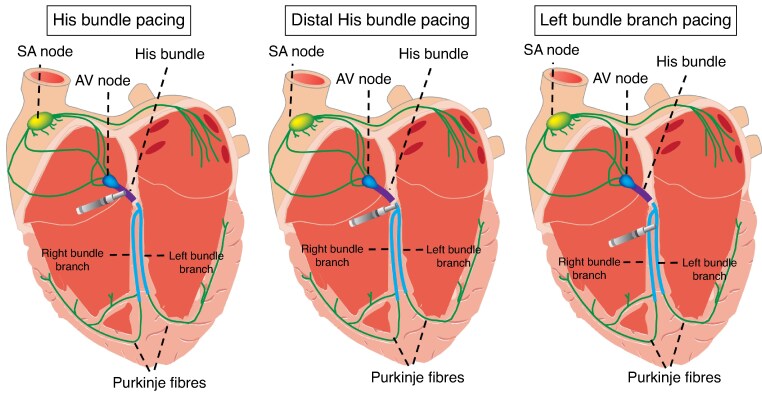
Mechanisms of conduction system pacing.

Conduction system pacing (CSP) has emerged as an alternative to RVP and BVP through recruitment of the innate conduction pathways.^9–15^ This approach results in greater physiological activation and cardiac synchrony to attain improved pacing and clinical outcomes.^16^ Current modalities for CSP include His bundle pacing (HBP), where leads are implanted in the His bundle, and left bundle branch area pacing (LBBAP), where leads are positioned in the interventricular septum to enable left bundle branch capture (*Figure 1*). The advancement of CSP implantation in addition to the growing body of evidence has led to the synthesis of recommendations and guidance surrounding CSP in recently released clinical guidelines. The 2023 HRS/APHRS/LAHRS guidelines on Cardiac Physiologic Pacing provided recommendations on patients undergoing pacemaker implant who are expected to require substantial ventricular pacing (≥20–40%) may be considered for Cardiac Physiologic Pacing CPP (this includes BIV or CSP) class 2a to reduce the risk of pacing-induced cardiomyopathy. It also recommended the use of CPP in patients with normal EF who will require substantial RV pacing class 2b.^17^ It is understandable that patients undergoing AVNA will require almost 100% pacing.

Studies have reported feasibility and improved electrocardiographic and echocardiographic outcomes for HBP when implemented with AVNA.^18,19^ Additionally, studies evaluating LBBAP and AVNA have also reported feasibility and positive outcomes corresponding to safety and threshold values.^20–22^ However, there are overall only few RCTs directly comparing CSP with BVP and therefore, limited robust evidence to immediately determine whether CSP could be an efficacious alternative to BVP for facilitating a pace and ablate strategy with AVNA. There is limited prospective and randomized data on CSP and AVNA that evaluating the efficacy of CSP with AVNA compared to BVP with only one prior meta-analysis primarily focused on HBP.^23^ Hence, we conducted an updated systematic review and meta-analysis to evaluate and compare clinical outcomes for patients receiving CSP or BVP, with AVNA.

## Methods

The Preferred Reporting Items for Systematic Reviews and Meta-Analyses (PRISMA) guidelines were adhered to in the conducting of this systematic review and meta-analysis. The systematic review protocol was published *a priori* on PROSPERO. The registration number is CRD42024510974.

### Data sources and searches

PubMed, EMBASE, and the Cochrane Library were searched from 1983 to 23 January 2024. The complete search strategies are fully described in the Supplementary Digital Content S1. The bibliographies of similar and related systematic reviewers also searched along with the reference sections of included studies. Only articles written in English were considered.

### Study selection

Randomized control trials, non-randomized control trials, case–control studies, cohort studies, retrospective studies, and cross-sectional studies that included patients with AF that underwent treatment with CSP and AVNA were eligible for this review. Studies that conveyed primary and/or secondary outcome variables in addition to the above were included. Four reviewers (A.M., A.M.A., K.A., I.K.) independently screened the retrieved articles for eligibility following deduplication by title and abstract. Any conflicts were resolved by discussion with a fifth reviewer (M.I.).

### Primary and secondary outcomes

The primary outcome was LVEF on follow-up and post-procedural QRS duration. Secondary outcomes included procedural success, periprocedural adverse events, pacing thresholds on implant and at follow-up, incidence of lead revision, New York Heart Association (NYHA) score on follow-up, incidence of HF associated hospitalization, and incidence of all-cause mortality.

### Data extraction and quality assessment

Two reviewers (A.M. and S.S.) independently extracted data from the included studies. Study characteristics collated included authors, year of study, county of study, study methodology, number and specific CSP modalities, and presence of control population. Sample population characteristics included sample size, mean age (SD), gender distribution, body mass index, prevalence of comorbidities, classification of AF [paroxysmal AF (PAF), persistent AF, or permanent AF], and previous AF management received (electrical cardioversion, catheter ablation). Baseline electrocardiographic and echocardiographic features were also collated in addition to the NYHA classification, NT-pro-BNP levels, and mean number of HF associated hospitalizations per patient. Primary outcomes collated included follow-up LVEF and QRS duration following device implantation. Secondary outcome data included periprocedural outcomes (procedural success, procedural time, fluoroscopy time, type of device implantation including any backup RV lead and atrial lead, and pacing parameters). Procedural details corresponding to unsuccessful implantations and periprocedural adverse events were recorded. Outcomes collated from follow-up data included LV end-diastolic volume index (LVEDVi), LV end-systolic volume index (LVESVi), NYHA, tricuspid regurgitation (TR) grade, and loop diuretic usage, and HF associated hospitalization were noted. Outcomes corresponding to AVNA such as time of AVNA relative to CSP implantation, procedural success, acute threshold increase, and loss of AV block were collated. Any discrepancies between two reviewers were arbitrated by a third reviewer (M.I.) and resolved by consensus. The Grading of Recommendations, Assessment, Development and Evaluation (GRADE) approach was applied to assess individual study methodological quality.^24^

### Risk of bias in individual studies

Risk of bias was assessed by 2 reviewers (A.M., R.C.) using the RoB 2 and ROBINS-I tool.^25,26^ Disparities between investigators were resolved following discussion with a third reviewer (M.I.) until consensus reached.

### Stata analysis

Outcome measures were continuous (QRSd, LVEF, NYHA, pacing thresholds) and binary (HF associated hospitalization and all-cause mortality). Data were analysed using a two-group comparison of continuous outcomes using random effects modelling with a restricted maximum likelihood method. Two group comparisons of binary outcomes were conducted with the Mantel–Haenszel model. Continuous were compared between patients who received CSP and BVP and were reported as a pooled mean difference with a 95% confidence interval (CI). Binary outcomes were reported as odds ratios with a 95% CI. Statistical heterogeneity was quantified using I^2^ statistic, where I^2^ > 60% was considered significant, moderate for I^2^ if between 30% and 60%, and a low if I^2^ < 30%. Substudy analysis was performed between CSP and BVP, HBP and BVP, LBBAP and BVP, and HBP and LBBAP. Analysis of heterogeneity was performed with sensitivity and subgroup analysis of the population. Publication bias was assessed using the Egger test and visually estimated with funnel plots. To explore potential sources of heterogeneity, meta-regression was applied to test the influence of mean age, male proportion, baseline LVEF, and baseline QRSd. Statistical analysis was performed with the Metan module in Stata Version 17 (© 2021 StataCorp LLC, USA).

## Results

### Literature search

**Figure 2:**
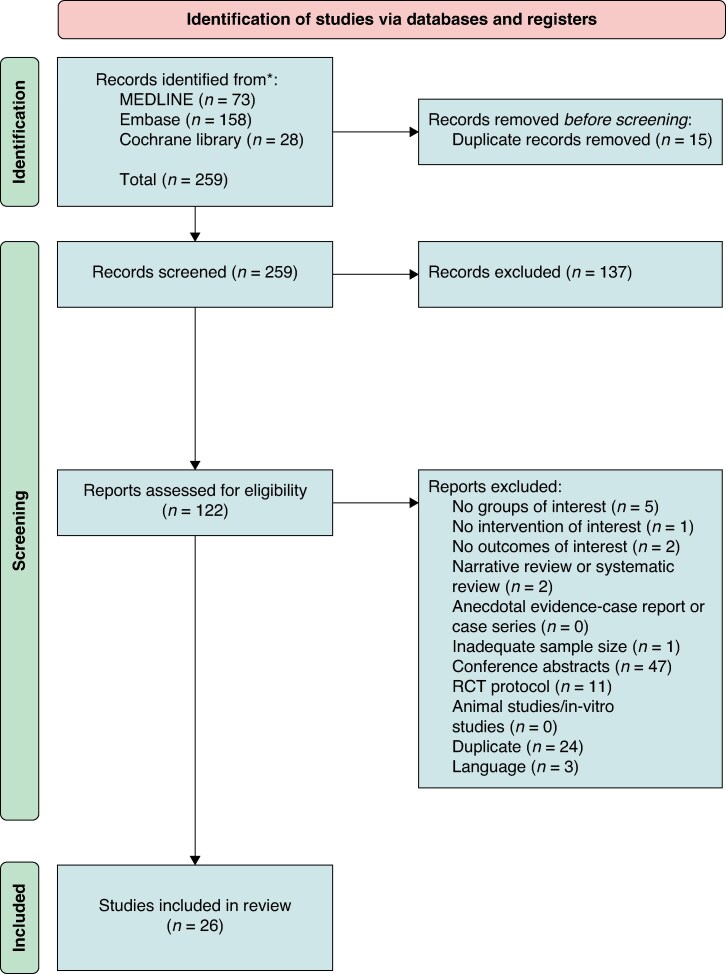
PRISMA flow diagram.

The search retrieved 259 studies results of which 15 duplicate records were manually removed. Following title and abstract review, 137 were excluded. Of the remaining 122 full text articles, 97 were excluded for the specified reasons illustrated in *Figure 2*. The remaining 26 studies were included.^18–22,27–46^ Five of the included studies were utilized for meta-analysis.^18,20,30,31,45^ The overall quality of the studies using the GRADE approach was moderate.

### Characteristics of the included studies

Detailed patient characteristics are presented in *Table 1*. Included studies were composed of 2 RCTs,^30,46^ 14 prospective studies,^18,22,27,29,35,37,39–45,47^ and 10 retrospective studies.^19–21,28,31–34,36,38^ The year of included studies ranged from 2006 to 2023. The total population across all studies were 2021 with 1652 and 369 patients receiving CSP and BVP with AVNA, respectively. A total of 1069 HBP implantations and 644 LBBAP implantations were conducted with 61 patients receiving both pacing modalities. The mean age range across studies was 63.6–79.2 years old while the male gender prevalence ranged from 28.0% to 76.0%. Further information pertaining to prevalence of commodities, AF classification and echocardiographic features are presented in *Tables 2* and *3.*

**Table 1:** Study characteristics

Study	Year	Country	Study methodology	Sample size	Number of patients with CSP implantation and AVNA	Number of patients with HBP implantation and AVNA	Number of patients with LBBAP implantation and AVNA	Number of patients with BVP implantation and AVNA
Palmisano, Parlavecchio, Crea *et al*. 2023	2023	Italy	Prospective observational study	119	119 (100)	34 (28.6)	85 (71.4)	0
Ivanovski *et al*. 2023	2023	Slovenia	Retrospective observational study	68	68 (100)	37 (54.4)	31 (45.6)	0
Liu *et al*. 2023	2023	USA	Retrospective observational study	24	24 (100)	0	24	0
Palmisano, Parlaveccio, Vetta *et al*. 2023	2023	Italy	Sub-analysis of prospective, multicentre, observational study	107	107 (100)	40 (37.4)	67 (62.6)	0
Palmisano, Ziacchi *et al*. 2023	2023	Italy	Sub-analysis of prospective, multicentre, observational study	373	110 (29.5)	68 (18.2)	42 (11.3)	263 (70.5)
Rijks *et al*. 2023	2023	The Netherlands	Prospective observational study	25	25 (100)	0	25 (100)	0
Qi *et al*. 2023	2023	China	Prospective observational study	31	31 (100)	22 (71.0)	9 (29.0)	0
Nam *et al*. 2023	2023	Australia	Retrospective observational study	8	8 (100)	8 (100)^a^		0
Ye *et al*. 2023	2023	China	Prospective observational study	33	33 (100)	33 (100)^a^		0
Cai *et al*. 2023	2022	China	Prospective observational study	99^b^ 172^c^	99 (100)^b^ 172^c^	86 (50)^c^	99 (100)^b^ 86 (50)^c^	0
Pillai *et al*. 2023	2022	USA	Retrospective observational study	98	98 (100)	48 (49.0)	50 (51.0)	0
Ivanovski *et al*. 2022	2022	Slovenia	Retrospective observational study	50	37 (74.0)	27 (54.0)	10 (20.0)	13 (26.0)
Zizek *et al*. 2022	2022	Slovenia	Prospective observational study	24	12 (50)	12 (50)	0	12 (50)
Huang *et al*. 2022	2022	China	Randomized controlled trial	50	25 (50)	25 (50)	0	25 (50)
Jin *et al*. 2022	2022	China	Retrospective observational study	56	56 (100)	0	56 (100)	0
Vijayaraman *et al*. 2022	2022	USA	Retrospective observational study	223	110 (49.3)	84 (37.7)^d^	46 (20.6)^d^	56 (25.1)
Chaumont *et al*. 2023	2022	France	Prospective observational study	83	83 (100)	83 (100)	0	0
Morina-Vazquez *et al*. 2021	2021	Spain	Prospective observational study	39	39 (100)	39 (100)	0	0
Wu *et al*. 2021	2021	China	Prospective observational study	178	178 (100)	130 (73.0)	48 (29.7)	0
Su *et al*. 2020	2020	China	Prospective observational study	94	94 (100)	94 (100)	0	0
Sun *et al*. 2020	2020	China	Prospective observational study	18	18 (100)	18 (100)	0	0
Wang *et al*. 2019	2019	China	Retrospective observational study	55	52 (94.5)	44 (84.6)^e^	8 (15.4)	0
Deshmukh *et al*. 2020	2018	USA	Retrospective observational study	13	13 (100)	13 (100)	0	0
Huang *et al*. 2017	2017	China	Prospective observational study	52	52 (100)	52 (100)	0	0
Vijayaraman *et al*. 2017	2017	USA	Retrospective observational study	42	42 (100)	42 (100)	0	0
Occhetta *et al*. 2006	2006	Italy	Randomized controlled (cross over) trial	18	18 (100)^f^	18 (100)^f^	0	0

CSP, conduction system pacing; AVNA, atrioventricular node ablation; HBP, His bundle pacing; LBBAP, left bundle branch area pacing; CP, conventional pacing; BVP, biventricular pacing.

^a^Received combination of HBP with LBBAP.

^b^Initial LBBAP study population.

^c^Matched population derived from retrospective data for subsequent comparisons with initial LBBAP study population.

^d^Twenty patients received both HBP and LBBAP implantation.

^e^Failed procedures.

^f^Received both HBP and right ventricular pacing.

**Table 2:** Conduction system pacing population demographics

Study	Specified intervention	Age	Gender-male prevalence	Body mass index	Comorbidities						AF classification		
					CAD	DM	HTN	CVA	CKD	COPD	PAF	Persistent AF	Permanent AF
Palmisano, Parlavecchio, Crea *et al*. 2023	Combined CSP	79.0	31.1	*Obesity prevalence—21.0	14 (11.8)	55 (46.2)	95 (79.8)	12 (10.1)	41 (34.4)	4 (3.36)	3 (2.52)	6 (5.04)	110 (92.4)
Ivanovski *et al*. 2023	Combined CSP	71 ± 8	42.6	N/A	14 (20.6)	18 (26.5)	49 (72.1)	N/A	N/A	N/A	N/A	N/A	N/A
Liu *et al*. 2023	LBBAP	78 ± 5	37	N/A	11 (46)	10 (42)	19 (79)	3 (13)	N/A	4 (17)	0 (0)	24 (100)	0
Palmisano, Parlaveccio, Vetta *et al*. 2023	Combined CSP	79.0 ± 9.1	33.6	N/A	11 (10.3)	54 (50.5)	85 (79.4)	12 (11.2)	42 (39.3)	4 (3.7)	0	0	107 (100)
Palmisano, Ziacchi *et al*. 2023	HBP	76.5 ± 8.0	48.5	Obesity prevalence—17 (25)	27 (39.7)	25 (36.8)	56 (82.4)	9 (13.2)	28 (41.2)	15 (22.1)	0 (0)	0	68 (100)
	LBBAP	77 ± 10.7	52.4	Obesity prevalence—12 (28.6)	18 (42.9)	15 (35.7)	34 (81)	6 (14.3)	17 (40.5)	11 (26.2)	0 (0)	0	42 (100)
Rijks *et al*. 2023	LBBAP	79.2 ± 4.2	40	27.0 ± 4.6	3 (12)	2 (8)	17 (68)	4 (16)	N/A	3 (12)	1 (4)	21 (84)	3 (12)
Qi *et al*. 2023	Combined CSP	74.6 ± 7.1	29.0	N/A	9 (29.0)	11 (12.1)	22 (70.1)	4 (12.9)	Creatinine—71.7 (62.1–79.8)	2 (6.5)	0	31 (100)	0
Nam *et al*. 2023	Combined CSP	72 ± 10	50.0	N/A	N/A	N/A	N/A	N/A	N/A	N/A	4 (50)	0 (0)	4 (50)
Ye *et al*. 2023	Combined CSP	68.5 ± 7.7	61.0	N/A	13 (39.4)	9 (27.3)	21 (63.6)	N/A	N/A	N/A	0	0	33 (100)
Cai *et al*. 2023	LBBAP	70.5 ± 9.5	51.2	N/A	58 (67.4)	26 (30.2)	60 (69.8)	N/A	22 (25.6)	N/A	0	86 (100)	0
	HBP	70.3 ± 10.4	55.8	N/A	63 (73.3)	20 (23.3)	52 (60.5)	N/A	23 (26.7)	N/A	0	86 (100)	0
Pillai *et al*. 2023	HBP	75.8 ± 7.9	60.4	N/A	15 (31.3)	9 (19)	34 (71)	N/A	N/A	N/A	0	48 (100)	0
	LBBAP	77.0 ± 6.7	28.0	N/A	17 (34)	8 (16)	31 (62)	N/A	N/A	N/A	0	50 (100)	0
Ivanovski *et al*. 2022	HBP	71 (62–75)	37.0	N/A	6 (22.2)	8 (29.6)	17 (63.0)	N/A	N/A	N/A	N/A	N/A	N/A
	LBBAP	69 (67–78)	70.0	N/A	4 (40)	2 (20)	8 (80)	N/A	N/A	N/A	N/A	N/A	N/A
Zizek *et al*. 2022	HBP	68.5 ± 6.8	41.7	N/A	3 (25)	5 (41.7)	8 (66.6)	N/A	N/A	N/A	N/A	N/A	N/A
Huang *et al*. 2022	HBP	63.6 ± 12.1	76.0	N/A	6 (24)	6 (24)	17 (68)	N/A	Creatinine—1.1 ± 0.3 mg/dL	N/A	N/A	25 (100)	N/A
Jin *et al*. 2022	LBBAP	75.3	53.6	24.303571	8 (14.3)	14 (25)	37 (66.1)	8 (14.3)	3 (5.36)	N/A	N/A	56 (100)	
Vijayaraman *et al*. 2022	Combined CSP	75 ± 10	55.0	29 ± 7	47 (45)	31 (28)	85 (77)	N/A	N/A	N/A	21 (19)	70 (63)	19 (17)
Chaumont *et al*. 2023	HBP	71 ± 12	51.0	28 ± 6	7 (8)	13 (16)	54 (65)	N/A	N/A	N/A	4 (5)	0	73 (88)
Morina-Vazquez *et al*. 2021	HBP	77 (70–81)	28.2	N/A	4 (10.3)	N/A	33 (84.6)	N/A	N/A	N/A	0	0	36 (92.3)
Wu *et al*. 2021	Combined CSP	69.3 ± 10.1	60.6	24 ±3.2	23 (13.5)	43 (25.3)	102 (60)	N/A	42 (24.7)	N/A	0	170 (100)	
Su *et al*. 2020	HBP	70.1 ± 10.5	57.4	*24.4 ± 3.8	*10 (12.3)	*15 (18.5)	N/A	N/A	*31 (38.3)	N/A	0	94 (100)	
Sun *et al*. 2020	HBP	72.2 ± 8.7	52.9	N/A	3 (17.6)	5 (29.4)	6 (35.3)	2 (11.8)	N/A	1 (5.9)	0	0	18 (100)
Wang *et al*. 2019	Combined CSP	67.6 ± 10.9	71.2	N/A	13 (25)	20 (38.5)	38 (73.1)	N/A	N/A	N/A	0	55 (100)	
Deshmukh *et al*. 2020	HBP + LV pacing	69.9 ± 10.7	76.9	N/A	N/A	7 (53.8)	13 (100)	N/A	6 (46.2)	N/A	N/A	N/A	N/A
Huang *et al*. 2017	HBP	72.8 ± 8.3	61.9	N/A	12 (28.6)	10 (23.8)	33 (78.6)	16 (38.1)	5 (11.9)	N/A	0	52 (100)	
Vijayaraman *et al*. 2017	HBP	74 ± 11	45.0	N/A	15 (36)	8 (19)	27 (64)	N/A	N/A	N/A	5 (12)	20 (48)	N/A
Occhetta *et al*. 2006	HBP	71.4 ± 5.6	50.0	N/A	5 (27.8)	N/A	7 (38.9)	N/A	N/A	N/A	N/A	N/A	N/A

CAD, coronary artery disease; DM, diabetes mellitus; HTN, hypertension; CVA, cerebrovascular accident; CKD, chronic kidney disease; COPD, chronic obstructive pulmonary disease; AF, atrial fibrillation; PAF, paroxysmal atrial fibrillation; CSP, conduction system pacing; LBBAP, left bundle branch area pacing; HBP, His bundle pacing; LV, left ventricular.

*From the 81 patients with successful HBP implantation.

**Table 3:** Conduction system pacing population baseline electrocardiographic and echocardiographic features

Study	Specified intervention	Electrocardiographic features				Echocardiographic features		NYHA classification		NT-pro-BNP	Number of HF related hospitalizations per patient	AF management		
		Baseline QRSd/ms	QRS > 130 ms	RBBB (%)	LBBB (%)	Baseline LVEF/%	LVEF < 50% (%)	Mean/median	Class III–IV (%)			Duration of persistent/permanent AF/months	Previous electrical cardioversions (%)	Previous catheter ablation (%)
Palmisano, Parlavecchio, Crea *et al*. 2023	Combined CSP	113.7	42 (35.3)	8 (6.72)	26 (21.8)	48.5	42 (35.3)	2.86	107 (89.2)	N/A	1.56	12.3	40 (33.6)	6 (5.04)
Ivanovski *et al*. 2023	Combined CSP	128 (110–140)	N/A	2 (2.94)	6 (8.82)	40 ± 15	N/A	3	54 (79.4)	2969 (1569–3635) pg/mL	N/A	N/A	N/A	N/A
Liu *et al*. 2023	LBBAP	117 ± 32	N/A	N/A	N/A	44 ± 14	N/A	N/A	N/A	N/A	N/A	N/A	N/A	N/A
Palmisano, Parlaveccio, Vetta *et al*. 2023	Combined CSP	114.7 ± 32.3	33 (30.8)	7 (6.5)	24 (22.4)	44.8 ± 11.8	EF < 40%—60 (56.1)	3.0 ± 0.5	92 (86.0)	N/A	1.6 ± 0.9	12 (6–18)	38 (35.5)	6 (5.6)
Palmisano, Ziacchi *et al*. 2023	HBP	95.0 ± 24.3	4 (5.9)	0 (0)	3 (4.4)	40.8 ± 12.0	EF < 40%—33 (48.5)	2.8 ± 0.7	47 (69.2)	N/A	1.5 ± 1.2	32.3 ± 38.6	41 (60.3	12 (17.6)
	LBBAP	112.6 ± 28.3	11 (26.2)	2 (4.8)	8 (19.0)	42.6 ± 10.9	EF < 40%—22 (52.4)	2.9 ± 0.7	31 (73.8)	N/A	1.5 ± 1.4	28.4 ± 30.6	28 (66.7)	4 (9.5)
Rijks *et al*. 2023	LBBAP	92 (87–98)	N/A	N/A	2 (8)	53.0 ± 7.0	N/A	N/A	N/A	N/A	N/A	N/A	N/A	13 (52)
Qi *et al*. 2023	Combined CSP	N/A	N/A	N/A	N/A	60.6 ± 12.1	N/A	N/A	N/A	253 (185–419.0) pg/mL	N/A	N/A	N/A	***2.3 ± 0.6
Nam *et al*. 2023	Combined CSP	118 ± 46	0 (0)	N/A	N/A	53.0 ± 4.00	0 (0)	N/A	N/A	N/A	N/A	N/A	N/A	3 (37.5)
Ye *et al*. 2023	Combined CSP	107.4 ± 27.2	N/A	9 (27.3)	0 (0)	59.2 ± 13.7	6 (50)	2.7 ± 0.6	N/A	N/A	N/A	N/A	N/A	N/A
Cai *et al*. 2023	LBBAP	111.7 ± 30.5	N/A	9 (10.5)	18 (20.9)	41.2 ± 15.0	EF < 40%—50 (58.1)	2.88	68 (79.1)	N/A	N/A	N/A	N/A	N/A
	HBP	111.4 ± 32.0	N/A	11 (12.8)	16 (18.6)	40.6 ± 14.8	EF < 40%—57 (66.3)	2.97	71 (82.6)	N/A	N/A	N/A	N/A	N/A
Pillai *et al*. 2023	HBP	97.9 ± 24	N/A	7 (14.6)	1 (2.1)	53.3 ± 9.4	N/A	N/A	N/A	N/A	N/A	N/A	N/A	N/A
	LBBAP	102.7 ± 20.5	N/A	4 (8)	1 (2)	53.1 ± 10.4	N/A	N/A	N/A	N/A	N/A	N/A	N/A	N/A
Ivanovski *et al*. 2022	HBP	100 ± 13	N/A	N/A	N/A	39 (30–45)	N/A	3	N/A	N/A	N/A	N/A	N/A	N/A
	LBBAP	105 ± 15	N/A	N/A	N/A	28 (20–42)	N/A	3	N/A	N/A	N/A	N/A	N/A	N/A
Zizek *et al*. 2022	HBP	91 ± 12	N/A	N/A	N/A	40 (37–45)	N/A	3	12 (100)	1616 (1010–2792) pg/mL	N/A	N/A	N/A	N/A
Huang *et al*. 2022	HBP	99.8 ± 15.3	N/A	N/A	N/A	31.9 ± 7.0	25 (100)	2.8 ± 0.6	N/A	**3.2 ± 0.6	N/A	N/A	N/A	N/A
Jin *et al*. 2022	LBBAP	90.42857143	N/A	N/A	N/A	53.0	N/A	3	N/A	N/A	N/A	N/A	N/A	N/A
Vijayaraman *et al*. 2022	Combined CSP	103 ± 25	N/A	12 (11)	11 (10)	47 ± 14	47 (43)	N/A	N/A	N/A	N/A	N/A	N/A	N/A
Chaumont *et al*. 2023	HBP	102 ± 22	*>120–10 (12)	6 (7)	3 (4)	47 ± 14	45 (56)	3.0 ± 0.7	54 (75)	N/A	N/A	N/A	N/A	N/A
Morina-Vazquez *et al*. 2021	HBP	N/A	N/A	N/A	N/A	55 (45–60)	N/A	N/A	20 (51.3)	N/A	N/A	N	N/A	N/A
Wu *et al*. 2021	Combined CSP	109.9 ± 30.1	N/A	N/A	38 (22.4)	34.3 ± 7.7	N/A	2.9 ± 0.6	N/A	606.7 pg/dL	N/A	N/A	N/A	N/A
Su *et al*. 2020	HBP	95.9 ± 12.7	N/A	N/A	N/A	45.3 + 14.9	N/A	2.78 ± 0.64	81 (100)	440 (209–972) pg/dL	N/A	N/A	N/A	N/A
Sun *et al*. 2020	HBP	91.1 ± 20.6	N/A	N/A	N/A	48.8 ± 11.2	N/A	N/A	N/A	N/A	N/A	7.0 ± 6.3	N/A	***1.8 ± 1.0
Wang *et al*. 2019	Combined CSP	96.4 ± 17.1	N/A	N/A	N/A	35.1 ± 11.7	<35%—42 (80.8)	N/A	34 (65.4)	N/A	N/A	N/A	N/A	N/A
Deshmukh *et al*. 2020	HBP + LV pacing	166.8 ± 19.1	N/A	N/A	8 (62)	28.1 ± 8.0	13 (100)	N/A	N/A	N/A	N/A	N/A	N/A	N/A
Huang *et al*. 2017	HBP	107.1 ± 25.8	N/A	N/A	N/A	44.9 ± 14.6	N/A	2.80	N/A	501.8 pg/mL	N/A	N/A	N/A	N/A
Vijayaraman *et al*. 2017	HBP	116 ± 28	N/A	N/A	N/A	43 ± 13	N/A	2.5 ± 5	N/A	N/A	N/A	N	N/A	N/A
Occhetta *et al*. 2006	HBP	88.3 ± 7.1	N/A	N/A	N/A	52.0 ± 9.1	6 (33.3)	2.4 ± 0.4	8 (33.3)	N/A	N/A	N/A	N/A	N/A

CAD, coronary artery disease; DM, diabetes mellitus; HTN, hypertension; CVA, cerebrovascular accident; CKD, chronic kidney disease; COPD, chronic obstructive pulmonary disease; AF, atrial fibrillation; PAF, paroxysmal atrial fibrillation; CSP, conduction system pacing; LBBAP, center bundle branch area pacing; HBP, His bundle pacing; LV, center ventricular.

*QRS > 120 ms.

**lgBNP concentration.

***Average number of ablations per patients.

### Procedural outcomes

A total of 1026 HBP procedures were successfully conducted (95.9%) while 630 cases of LBBAP were successfully performed (97.8%) (*Table 4*). A total of 49 cases were converted from an initially intended HBP implantation to LBBAP, while 10 cases of planned LBBAP were converted to left septal pacing (LSP). Backup RV lead insertion was documented in 871 cases while atrial lead implantation was conducted in 306 cases. CSP implantation procedural time ranged from 34 to 120 min while fluoroscopy duration ranged from 4.5 to 18 min. Conduction system pacing was facilitated through a range of devices ranging from single chamber PPM to biventricular ICD (*Table 4*). Procedural pacing parameters corresponding to pacing threshold, impedance, QRS duration, and R wave characteristics are presented in *Table 5*. Documented reasons for unsuccessful HBP and LBBAP implantation failure are presented in *Table 6*. Acute periprocedural adverse events were documented in 23 cases of CSP implantation (2.15%) with no events of periprocedural mortality observed (*Table 6*). Acute threshold elevation was observed in 34 cases of HBP implantation (3.22%) while none were noted in the LBBAP pooled cohort (*Table 6*). Lead repositioning following acute threshold changes was required in six cases (0.69%).

**Table 4:** Conduction system pacing periprocedural details and outcomes

Study	Specified intervention	Successful implantation—HBP (%)	Selective HBP (%)	Successful implantation—LBBAP (%)	Selective LBBAP capture (%)	Conversion of HBP attempt to LBBAP (%)	CSP implantation procedural time/min	CSP implantation fluoroscopy duration/min	Type of device implanted					Backup RV lead (%)	Atrial lead implantation (%)
									Single chamber PPM (%)	Dual chamber PPM (%)	Biventricular PPM (%)	Dual chamber ICD	Biventricular ICD		
Palmisano, Parlavecchio, Crea *et al*. 2023	Combined CSP	34 (100)	N/A	85 (100)	N/A	0	Implantation time— 61.4 Total procedure time—86.0	Implantation—8.65 Overall—11.5	0	26 (21.8)	73 (61.3)	6 (5.04)	14 (11.8)	109 (100)	87 (73.1)
Ivanovski *et al*. 2023	Combined CSP	37 (100)	N/A	31 (100)	N/A	0	N/A	6 (4.2–8.1)	N/A	N/A	N/A	10 (14.7)	N/A	0 (0)	1 (1.47)
Liu *et al*. 2023	LBBAP	N/A	N/A	22 (91.7)	20 (84.2) 1—non-selective HBP 1—deep septal pacing	N/A	N/A	10.2 ± 4.1	0	0	18 (75)—4 received for CRT purposes, 14 received backup RV lead in case of LBBAP failure	0	6 (25)	14 (58)	0
Palmisano, Parlaveccio, Vetta *et al*. 2023	Combined CSP	40 (100)	N/A	67 (100)	N/A	0	N/A	N/A	0	31 (29)	59 (55.1)	4 (3.7)	13 (12.1)	79 (73.8)	73 (68.2)
Palmisano, Ziacchi *et al*. 2023	HBP	68 (100)	N/A	N/A	N/A	0	65.9 ± 26.5—compared to BVP—*P* < 0.001	17.3 ± 22.2—compared to BVP—*P* = 0.096	0	26 (56.5)	20 (43.5)	11 (50)	11 (50)	68 (100)	24 (27.9)
	LBBAP	N/A	N/A	42 (100)	N/A	N/A	56.0 ± 20.5—compared to BVP—*P* < 0.001	10.2 ± 7.4—compared to BVP—*P* = 0.003	0	33 (97.1)	2 (5.9)	5 (62.5)	3 (37.5)	26 (61.9)	16 (38.1)
Rijks *et al*. 2023	LBBAP	N/A	N/A	25 (100)	18 (72)	N/A	N/A	N/A	N/A	N/A	N/A	N/A	N/A	N/A	N/A
Qi *et al*. 2023	Combined CSP	22 (100)	N/A	9 (100)*	N/A	9 (29)	N/A	15.5 ± 4.9	0	0	31 (100)	0	0	31 (100)	0
Nam *et al*. 2023	Combined CSP	8 (100)	N/A	8 (100)—DsLBBAP	N/A	0	N/A	N/A	0	4 (50)	4 (50)	0	0	0	4 (50)
Ye *et al*. 2023	Combined CSP	30 (90.9)	N/A	31 (93.9)	N/A	N/A	N/A	6.2 ± 1.0	0	33 (100)	0	0	0	0	0
Cai *et al*. 2023	HBP	86 (100)** 176 (81.9)	N/A	N/A	N/A	23	***136 (97.8–180) *P* = 0.021	N/A	0	19 (22.1)	19 (22.1)	7 (8.1)	41 (47.7)	36 (41.9)	N/A
	LBBAP	N/A	N/A	99 (100)	N/A	N/A	***120 (100–151.5) *P* = 0.021	N/A	0	39 (45.5)	4 (4.7)	1 (1.2)	41 (47.7)	3 (3.5)	N/A
Pillai *et al*. 2023	HBP	48 (100)	20 (44.4)	N/A	N/A	N/A	44 ± 24	16 ± 18 *P* < 0.01	0	26 (54.2)	13 (27.1)	2 (4.2)	0	RV lead—23 (47.9) CS lead—5 (10.4)	10 (20.8)
	LBBAP	N/A	N/A	50 (100)	N/A	N/A	34 ± 16	7 ± 6 *P* < 0.01	0	28 (56)	2 (4)	0 (0)	1 (2)	RV lead—0 CS lead—1 (2)	28 (56)
Ivanovski *et al*. 2022	HBP	27 (100)	11 (40.7)	N/A	N/A	N/A	N/A	6 (4.5–10) *P* < 0.001—comparing both with BIV	N/A	N/A	N/A	2 (7.41)	N/A	27 (100)	1 (3.70)
	LBBAP	N/A	N/A	10 (100)	10 (100)	N/A	N/A	4.5 (3.1–7.5) *P* < 0.001—comparing both with BIV	N/A	N/A	N/A	3 (30)	N/A	0	0
Zizek *et al*. 2022	HBP	12 (100)	5 (41.7)	N/A	N/A	N/A	N/A	7.0 ± 3.8 Compared to BiV (*P* < 0.0001)	0	0	12 (100)	0	0	12 (100)	0
Huang *et al*. 2022	HBP	50 (100)	N/A	N/A	N/A	1 (2)	N/A	N/A	0	0	20 (40)	0	30 (60)	50 (100)	N/A
Jin *et al*. 2022	LBBAP	N/A	N/A	46 (82.1)	N/A	LBBAP to LSP—10 (17.9)	N/A	N/A	N/A	N/A	N/A	N/A	N/A	0	N/A
Vijayaraman *et al*. 2022	Combined CSP	84 (100)	N/A	46 (100)	N/A	N/A	130 ± 67	17 ± 12	0	62 (56) ****	39 (36) ****	62 (56) ****	39 (36) ****	54 (49.1)	N/A
Chaumont *et al*. 2023	HBP	75 (90)	57 (76)	N/A	N/A	N/A	63 ± 26	6.4 ± 7.7	58 (76)	13 (18)	N/A	4 (5)	N/A	8 (11)	5 (7)
Morina-Vazquez *et al*. 2021	HBP	36 (92.3)	8 (20.5)	N/A	N/A	N/A	N/A	N/A	N/A	N/A	N/A	N/A	N/A	N/A	N/A
Wu *et al*. 2021	Combined CSP	106 (81.5)	91 (70)	64 (100)	40 (62.5)	16 (12.3)	N/A	N/A	0 (0)	CSP—39 (22.9)	42 (24.7)	0 (0)	89 (52.4)	131 (77.1)	39 (22.9)
Su *et al*. 2020	HBP	Acute success - 89 (94.7) Permanent success - 81 (86.2)	81 (86.2)	N/A	N/A	N/A	N/A	N/A	0 (0)	19 (23.5)	26 (32.1)	11 (13.5)	25 (30.9)	RV port—10 (12.3) LV port—71 (87.7)	10 (12.3)
Sun *et al*. 2020	HBP	17 (94)	5 (29.4)	N/A	N/A	N/A	99.4 ± 16.4	7.0 ± 2.6	0	18 (100)	0	0	0	18 (100)	0
Wang *et al*. 2019	Combined CSP	44 (93.6)	N/A	8 (72.7)	N/A	N/A	N/A	N/A	0	0	0	13 (25)	39 (75)	LV backup lead—31 (59.6)	8 (15.4)
Deshmukh *et al*. 2020	HBP + LV pacing	13 (100)	N/A	N/A	N/A	N/A	N/A	N/A	0	0	0	0	21 (100)	21 (100)	0
Huang *et al*. 2017	HBP	42 (80.8)	Direct His pacing—38 (90.5) Para-Hisian pacing—4 (9.52)	N/A	N/A	N/A	N/A	N/A	0	17 (40.5)	17 (40.5)*****	8 (19.0)	17 (40.5)*****	42 (100)	0
Vijayaraman *et al*. 2017	HBP	40 (95)	13 (33)	N/A	N/A	N/A	N/A	N/A	6 (14)	17 (40)	15 (36)	2 (5)	2 (5)	14 (36.8)	N/A
Occhetta *et al*. 2006	HBP	17 (94.4)	N/A	N/A	N/A	N/A	N/A	18 ± 9	0	18 (100)	0	0	N/A	18 (100)	N/A

HBP, His bundle pacing; LBBAP, left bundle branch associated pacing; CSP, conduction system pacing; PPM, permanent pacemaker; ICD, implantable cardioverter defibrillator; RV, right ventricle; CRT, cardiac resynchronization therapy; DsLBBAP, deep septal left bundle branch associated pacing; CS, coronary sinus; BiV, biventricular; AVNA, atrioventricular node ablation; LV, left ventricle; N/A, not applicable/available. *Following unsuccessful HBP. **Matched retrospective comparison. *** Total procedural time including AVN ablation ****Both PPM and ICD. *****Both BiV PPM and ICD.

**Table 5:** Pacing parameters on implantation

Study	Pacing threshold/V		Pacing impedance/Ohms Ω		QRS during implantation/ms		R wave/mV		AVNA		
	HBP	LBBAP	HBP	LBBAP	HBP	LBBAP	HBP	LBBAP	Performed at time of implantation (%)	Time between implantation and ablation/days	Procedural success (%)
Palmisano, Parlavecchio, Crea *et al*. 2023	1.24	0.54	560.9	673.3	N/A	N/A	N/A	N/A	119 (100)	N/A	119 (100)
Ivanovski *et al*. 2023	N/A	N/A	N/A	N/A	N/A	N/A	N/A	N/A	Unspecified	N/A	68 (100)
Liu *et al*. 2023	N/A	0.8 ± 0.3	N/A	710 ± 216	N/A	123 ± 14	N/A	9.9 ± 3.9	22 (91.7)	N/A	N/A
Palmisano, Parlaveccio, Vetta *et al*. 2023	1.2 ± 0.7	0.6 ± 0.5	555.2 ± 146.9	663.7 ± 161.5	111.3 ± 19.5*	111.3 ± 19.5*	N/A	N/A	97 (94.2)	17 (3–22)	N/A
Palmisano, Ziacchi *et al*. 2023	1.2 ± 0.7 Compared to BVP, *P* = 0.791	0.6 ± 0.4 Compared to BVP, *P* < 0.01	557.2 ± 147.8 Compared to BVP, *P* < 0.001	664 ± 162.1 Compared to BVP, *P* = 0.044	110.1 ± 19.5 Compared to BVP, *P* < 0.001	112.1 ± 17.4 Compared to BVP, *P* = 0.588	N/A	N/A	HBP—64 (94.1) Compared to BVP—*P* < 0.01 LBBAP—41 (97.6) Compared to BVP—*P* < 0.001	HBP—1.1 ± 4.9 Compared to BVP—*P* < 0.001 LBBAP—0.6 ± 4.0 Compared to BVP—*P* < 0.001	HBP—67 (98.5) Compared to BVP—*P* = 0.812 LBBAP—42 (100) Compared to BVP—*P* = 0.368
Rijks *et al*. 2023	N/A	0.5 (0.5–0.5)	N/A	552 ± 116	N/A	N/A	N/A	14 (9–19)	22 (88)	4–6 weeks following implantation	N/A
Qi *et al*. 2023	1.14 ± 0.45	0.77 ± 0.30	711.8 ± 197.8*	711.8 ± 197.8*	114.8 ± 12.1 Compared to intrinsic QRS *P* < 0.001	111.7 ± 11.0	N/A	N/A	30 (96.8)	Unspecified	28 (93.3)
Nam *et al*. 2023	1.0 ± 0.6	0.5 ± 0.2	552 ± 57	814 ± 171	101 ± 20	119 ± 17	2.4 ± 1,1	14 (9–19)	0 (0)	At least one month after	8 (100)
Ye *et al*. 2023	0.8 ± 0.3	0.7 ± 0.2	449.5 ± 77.6	567.8 ± 169	105.7 ± 13.2	117.8 ± 15.3	3.94 ± 3.04	14 (9–19)	N/A	N/A	N/A
Cai *et al*. 2023	1.09 ± 0.7	0.46 ± 0.15	N/A	537.3 ± 103.5	122.7 ± 18.9	130.8 ± 13.2	3.2 ± 3.1	11.4 ± 4.4	172 (100)	N/A	172 (100)
Pillai *et al*. 2023	1.29 ± 1.03	0.68 ± 0.27	546.8	N/A	119.6 ± 19.2	122 ± 8.4	5.3 ± 4	9.8 ± 5.5	LBBAP—50 (100) HBP—45 (93.8)	N/A	LBBAP—50 (100) HBP—45 (93.8) *P* = 0.11
Ivanovski *et al*. 2022	1.25 (1–2)	0.8 (0.5–1.1)	526 ± 87	750 ± 77	105 ± 17	127 ± 13	N/A	N/A	HBP—27 (100) LBBAP—10 (100)	N/A	HBP—27 (100) LBBAP—10 (100)
Zizek *et al*. 2022	1.55 (1.07–2.00)	N/A	548 (490–598)	N/A	95 ± 15	N/A	N/A	N/A	12 (100)	N/A	12 (100)
Huang *et al*. 2022	0.9 ± 0.6	N/A	515.1 ± 66.0	N/A	107.6 ± 12.5	N/A	4.2 ± 5.0	N/A	50 (100)	N/A	50 (100)
Jin *et al*. 2022	N/A	0.8 (0.5–1.00)	N/A	761 ± 191.0	N/A	109 (105–117)	N/A	11.8 ± 3.7	56 (100)	N/A	56 (100)
Vijayaraman *et al*. 2022	1.11 ± 0.7	0.73 ± 0.22	N/A	N/A	122 ± 21	128 ± 14	N/A	N/A	103 (94)	Remaining majority under 2 months	110 (100)
Chaumont *et al*. 2023	1.2 ± 0.7	N/A	486 ± 148	N/A	106 ± 17	N/A	3.9 ± 2.1	N/A	10 (13)	N/A	70 (93)
Morina-Vazquez *et al*. 2021	1.25 (0.95–2)	N/A	506 (350–600)	N/A	N/A	N/A	2.4 (1.4–4.2)	N/A	0 (0)	At least 15 days after implantation	36 (100)
Wu *et al*. 2021	1.01	0.465	N/A	N/A	96.78113208	112.975	2.88	11.2875	170 (100)	N/A	170 (98.3)
Su *et al*. 2020	1.0 ± 0.7	N/A	489.5	N/A	101.7 ± 20.4	N/A	2.6	N/A	81 (93.1)	N/A	87 (97.8)
Sun *et al*. 2020	1.1 ± 0.4	N/A	645.5 ± 133.7	N/A	114.9 ± 20.8	N/A	5.20 ± 4.10	N/A	17 (100)	N/A	17 (94)
Wang *et al*. 2019	Not specified	N/A	N/A	N/A	N/A	N/A	N/A	N/A	52 (100)	N/A	52 (98.1)
Deshmukh *et al*. 2020	2.2 ± 1.6	N/A	N/A	N/A	150 ± 22	N/A	N/A	N/A	13 (100)	N/A	13 (100)
Huang *et al*. 2017	1.5 ± 1.0	N/A	N/A	N/A	105.3 ± 23.9	N/A	N/A	N/A	42 (100)	N/A	42 (91.3)
Vijayaraman *et al*. 2017	1.0 ± 0.8	N/A	544 ± 125	N/A	127 ± 15	N/A	6.0 ± 5.9	N/A	30 (71.4)	8—(1–15) months 4—(1–12) years	40 (97.6)
Occhetta *et al*. 2006	0.92 ± 0.7	N/A	614 ± 177	N/A	121.1 ± 9.9	N/A	6.9 ± 3.4	N/A	18 (100)	N/A	18 (100)

HBP, His bundle pacing; LBBAP, left bundle branch associated pacing; CSP, conduction system pacing; N/A, not applicable/available; BVP, biventricular pacing. *Averaged across both HBP and LBBAP.

**Table 6:** Procedural details for unsuccessful implantation and periprocedural adverse events

Study	Specified intervention	Number of unsuccessful HBP implantations (%)	Documented reasons for HBP implantation failure	Number of unsuccessful LBBAP implantations (%)	Documented reasons for LBBAP implantation failure	Acute periprocedural adverse events	AVNA adverse events/complications	Acute high thresholds following AVNA (%)	Lead repositioning requirement following acute high thresholds (%)
Palmisano, Parlavecchio, Crea *et al*. 2023	CSP (HBP or LBBAP)	0	N/A	0	N/A	1 (0.84)—vascular access complication—groin haematoma—treated conservatively—extended hospital stay by 2 days	12 (10.1)—unsuccessful from axillary/subclavian vein approach—had to convert to femoral 1 (0.84)—unsuccessful from typical femoral approach—had to convert to left sided approach via femoral artery	1 (0.84)	1 (0.84)
Ivanovski *et al*. 2023	CSP (HBP or LBBAP)	0	N/A	0	N/A	None specified	None specified	None specified	None specified
Liu *et al*. 2023	LBBAP	N/A	N/A	2 (8.30)	2 (8.30)—unspecified	1 (4.17)—pocket haematoma—no intervention required	3 (13.6)—femoral access required	None specified	None specified
Palmisano, Parlaveccio, Vetta *et al*. 2023	CSP (HBP or LBBAP)	0	N/A	0	N/A	0	None specified	None specified	None specified
Palmisano, Ziacchi *et al*. 2023	CSP (HBP or LBBAP)	0	N/A	0	N/A	0	HBP: 1 (1.47)—femoral artery used for retrograde aortic approach 1 (1.47)—stable AV block not successful on first attempt and subsequent second ablation required	HBP—2 (2.94)	HBP—2 (2.94)—reposition required—no reoperation however or increased length of stay
Rijks *et al*. 2023	LBBAP	N/A	N/A	0	N/A	2 (9.09)—lead stability concerns due to lead dislocation during slitting—required immediate repositioning of the LBBAP lead	1 (4.54)—AVNA delayed due to back pain	Unspecified	Unspecified
Qi *et al*. 2023	CSP (HBP or LBBAP)	9 (29.0)	−3 (9.68)—difficulty recording the His bundle potential—switched to LBBAP −6 (19.4)—Unable to achieve acceptable HBP parameters—switched to LBBAP	0	N/A	HBP: 1 (3.22)—septal lead perforation during procedure however lead successfully re-implanted 1 (3.22)—mild pocket haematoma—on dual antiplatelet agents—resolved within one week with reinforced elastic compression badges 3 (9.66)—transient right bundle branch block—resolved prior to discharge	1 (3.23)—unable to achieve AV block	Unspecified	Unspecified
Nam *et al*. 2023	HBP with DsLBBAP	0	N/A	0	N/A	0	0	0	0
Ye *et al*. 2023	HBP + LBBAP	3 (9.09)	1 (3.03)—HBP fixation failure 2 (6.06)—high HBP thresholds during the procedure with resultant procedure abandonment	2 (6.06)	1 (3.03)—unsuccessful LBBP lead fixation due to severe TR 1 3.03)—unsuccessful LBB capture—instead had LVSP implanted	0	Unspecified	Unspecified	Unspecified
Cai *et al*. 2023	CSP (HBP or LBBAP)	32 (14.9)	11 (5.12)—increased threshold > 1 V at 0.5 ms following AVJ ablation 17 (7.91)—high capture/corrective thresholds (>2 V at 0.5 ms) 2 (0.93)—failure to correct LBBB 2 (0.93)—failure to achieve His fixation	0 (0)	N/A	0	HBP: 7 (3.26)—failure to attain 3rd degree AV block	Unspecified	Unspecified
Pillai *et al*. 2023	CSP (HBP or LBBAP)	0	N/A	0	N/A	0	HBP: 8 (16.7)—acute rise in threshold—V > 1 IN 2 cases and V > 2 in 6 cases. 4 (8.33)—exit block resulting in HBP extraction in 3 (1 acute, 2 chronic) and HBP deactivation in 1 3 (6.25)—failed initial attempt required 4 additional AVJ ablation procedure 5 (10.4)—femoral arterial access for retrograde aortic approach	HBP: 8 (14.5)	1 (2.08)—HBP extraction
Ivanovski *et al*. 2022	CSP (HBP or LBBAP)	0	N/A	0	N/A	0	1 (3.70)—acute rise in HBP lead threshold after AVNA—lead revision was not required	1 (3.70)	1 (3.70)
Zizek *et al*. 2022	HBP	0	N/A	N/A	N/A	0	1 (8.33)—acute rise in HBP lead threshold after AVNA—lead revision was not required	1 (8.33)	1 (8.33)
Huang *et al*. 2022	HBP	0	N/A	N/A	N/A	0	Unspecified	Unspecified	Unspecified
Jin *et al*. 2022	LBBAP	N/A	N/A	10 (17.9)	10 (17.9)—failure for lead positioning and instead received LVSP	2 (4.34)—incomplete RBBB—transient—resolved prior to discharge 1 (2.17)—PPM pocket haematoma—bleeding stopped with compression bandage and the haematoma was absorbed spontaneously	0	Unspecified	Unspecified
Vijayaraman *et al*. 2022	CSP (HBP or LBBAP)	0	N/A	0	N/A	0	0	Unspecified	Unspecified
Chaumont *et al*. 2023	HBP	8 (9.64)	8 (9.64)—inability to map the HB or attain satisfactory HBC thresholds during the implantation procedure (>2 V at 1 ms)	N/A	N/A	0	12 (16.0)—modulation of AV nodal conduction required 5 (6.67)—AVNA failure—reason unspecified	11 (14.7)	0
Morina-Vazquez *et al*. 2021	HBP	3 (7.70)	3 (7.7)—unable to fix pacing lead	N/A	N/A	0	0	0	0
Wu *et al*. 2021	CSP (HBP or LBBAP)	21 (16.5)	HBP: 19 (15.0)—inadequate His capture threshold 2 (1.57)—failed HBP lead fixation	0	N/A	0	HBP: 3 (2.31)—AVNA—reason unspecified	Unspecified	Unspecified
Su *et al*. 2020	HBP	5 (5.30)	1 (1.06)—inability to fix lead 4 (4.24)—high His capture thresholds (>4 V/0.5 ms) 2 (2.12)—injury of His bundle following ablation subsequently affecting His bundle pacing	N/A	N/A	0	8 (8.48)—injury to His bundle by ablation (increased threshold > 1/0.5 ms in two of the patients)—disruption of His bundle pacing 2 (2.12)—failed AVN ablation—reason unspecified	2 (2.12)	*His bundle injury
Sun *et al*. 2020	HBP	1 (5.56)	1 (5.56)—His bundle could not be mapped using a catheter—failure in AVN	N/A	N/A	0	1 (5.56)—His bundle could not be mapped using a catheter—failure in AVN 1 (5.56)—required AVN ablation from left side under aortic valve due to inability to achieve AVN from right sided access	0	0
Wang *et al*. 2019	CSP (HBP or LBBAP)	2 (4.35)	2 (4.35)—high His pacing thresholds—instead received BVP	0	N/A	0	1 (1.82)—failed AVN ablation—reason unspecified—received single chamber ICD and rate control medications	Unspecified	Unspecified
Deshmukh *et al*. 2020	HBP	0	N/A	N/A	N/A	0	0	3 (23.1)—rise of His capture thresholds more than 1 V	0
Huang *et al*. 2017	HBP	2 (4.55)	2 (3.8)—His bundle potential not recorded 2 (3.8)—His bundle injury from ablation	N/A	N/A	8 (16.0)—transient rise in RBBB—7 patients resolved during procedure 2 (4.00)—transient 3rd degree AV block during His bundle lead implantation—recovered during procedure	2 (3.8)—failed AVN ablation—unspecified reason 2 (3.8)—His bundle injury from ablation	Unspecified	Unspecified
Vijayaraman *et al*. 2017	HBP	2 (4.76)	1 (2.38)—difficulty in achieving thresholds 1 (2.38)—dislodgement of the HBP lead following ablation attempts	N/A	N/A	0	1 (1.19)—AVNA unsuccessful from right side—changed to left sided approach—threshold acutely increased from 1 to 3 V/1 ms and returned to initial values by end of procedure	5 (13.5)	0
Occhetta *et al*. 2006	HBP	1 (5.60)	1 (5.60)—lead instability due to AV junction anatomical changes due to previous mitral and aortic valve replacement	N/A	N/A	1 (5.6)—cardiac arrest (VF) 5 days following procedure—device explanted and a rate responsive single chamber ICD inserted—aetiology for VF arrest not specified	0	Unspecified	Unspecified

HBP, His bundle pacing; LBBAP, left bundle branch associated pacing; CSP, conduction system pacing; N/A, not applicable/available; BVP, biventricular pacing; AVNA, atrioventricular node ablation; LBBP, left bundle branch pacing; TR, tricuspid regurgitation; AVJ, atrioventricular junction; LVSP, left ventricular septal pacing.

Atrioventricular node ablation was successfully implemented in 1594 patients (99.1%) with 1333 cases specified to have been performed at the time of CSP implantation (84.6%). For cases with AVNA not performed during CSP implantation, the time interval between procedures generally ranged from 2 to 6 weeks.

A total of 369 patients were specified to have successfully received BVP. Fluoroscopy duration ranged from 14 to 23.5 min.

### Improvement in LVEF

**Figure 3:**
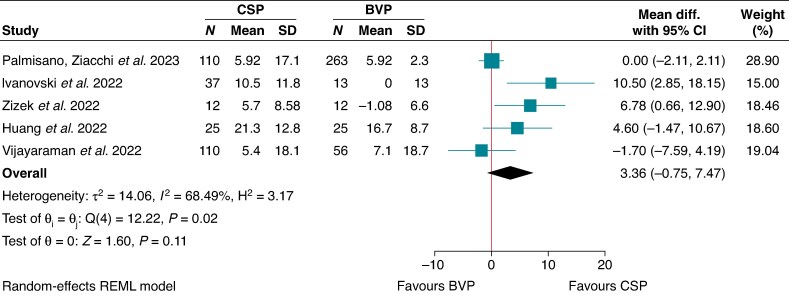
Change in LVEF - CSP vs. BVP.

Conduction system pacing demonstrated a non-significant improvement in LVEF compared to BVP by ∼3.36% (MD 3.36, 95% CI −0.75–7.47 I^2^ = 68.5; *P* = 0.11)^18,20,30,31,45^ (*Figure 3*). Overall, the rest of the included studies that were not incorporated in the meta-analysis also demonstrated an improvement in LVEF on follow-up (*Table 7*, Supplementary material online, *Table S4*). The improvement in LVEF was even more accentuated in studies that reported specific patient populations with impaired LVEF.^19,21,27–29,35,41,42^ Meta-analysis for this specific cohort of LV impairment could not be pursued as majority of these studies did not include a comparator BVP cohort.

**Table 7:** Conduction system pacing—outcomes on follow-up

Study	Specified intervention	F/U period	Baseline LVEF (%)	F/U—LVEF%	F/U—LVEDVi	F/U—LVESVi	Baseline—NYHA	F/U—NYHA	F/U—NT-Pro-BNP	HF hospitalizations	Mortality
Palmisano, Parlavecchio, Crea *et al*. 2023	Combined CSP	12 months (5–17)	48.5	49.9 ± 7.6	N/A	N/A	N/A	N/A	N/A	2 (1.68)	N/A
Ivanovski *et al*. 2023	Combined CSP	16 months (6–27)	40 ± 15	50.17647059	SR group—53 (49–57) NSR group—63 (47–82)	SR group—25 (25–26) NSR group—29 (21–46)	SR = 3.5 (3–4) NSR—3 (3–3)	SR—1.5 (1–2) NSR—2 (1–2)	SR—1437 (1042–2229) NSR—2034 (976–3001)	N/A	HBP—3 (8.11) LBBAP—1 (3.23)
Liu *et al*. 2023	LBBAP	3 months	44 ± 14	46 ± 12	N/A	N/A	N/A	N/A	N/A	N/A	N/A
Palmisano, Parlaveccio, Vetta *et al*. 2023	Combined CSP	12 (5–17) months	44.8 ± 11.8	49.9 ± 7.6	N/A	N/A	3.0 ± 0.5	N/A	N/A	3 (2.8)	N/A
Palmisano, Ziacchi *et al*. 2023	HBP	12 months	40.8 ± 12.0	46.8 ± 12.8	N/A	N/A	N/A	N/A	N/A	1 (1.5)	N/A
	LBBAP		42.6 ± 10.9	48.4 ± 12.3	N/A	N/A	N/A	N/A	N/A	1 (2.4)	N/A
Rijks *et al*. 2023	LBBAP	81 (47–235) days	53.0 ± 7.0	N/A	N/A	N/A	N/A	N/A	N/A	N/A	N/A
Qi *et al*. 2023	Combined CSP	6 months	60.6 ± 12.1	63.7 ± 5.8	46.0 ± 4.0 mm*	N/A	N/A	N/A	152 (92–248) pg/mL	N/A	HBP—0 LBBAP—0
Nam *et al*. 2023	Combined CSP	121 ± 92 days	53.0 ± 4.00	N/A	N/A	N/A	N/A	N/A	N/A	0	N/A
Ye *et al*. 2023	HBP	12 months	59.2 ± 13.7**	52.6 ± 5.24	N/A	136.1 ± 12.4	2.7**	2.1	N/A	N/A	N/A
	LBBAP			57.3 ± 11.2	N/A	140.5 ± 8.39		2	N/A	N/A	
Cai *et al*. 2023	HBP	28.9 ± 11.9 months	41.2 ± 15.0	1 year—52.7 ± 14.7 2 years—54.6 ± 14.8 3 years—57.1 ± 13.9	N/A	N/A	64 (79.0)***	1 year—NYHA III–IV—11 (13.6) 2 years—NYHA III–IV—9 (12.9) 3 years—NYHA III–IV—5 (14.3)	N/A	6 (6.98)	10 (11.6)
	LBBAP		40.7 ± 14.8	1 year—54.9 ± 12.8 2 years—54.7 ± 13.3 3 years—58.0 ± 11.4	N/A	N/A	63 (77.8)***	1 year—NYHA III–IV—12 (14.8) 2 years—NYHA III–IV—8 (11.4) 3 years—NYHA III–IV—3 (8.57)	N/A	9 (10.5)	5 (5.81)
Pillai *et al*. 2022	HBP	36 months	Overall—53.3 ± 9.4 38****	HBP—55 47****	CSP overall—44 ± 7 mm *P* = 0.09 49 ± 10 mm**** P = 0.84	CSP overall—32 ± 9 mm *P* = 0.93 39 ± 10 mm**** P = 0.62	N/A	N/A	N/A	N/A	7 (14.6)
	LBBAP		Overall −53.1 ± 10.4 38****	LBBAP—54 45****			N/A	N/A	N/A	N/A	1 (2)
Ivanovski *et al*. 2022	HBP	6 (5–13) months	39 (30–45)	49 (42–58)	61 ± 18 mL/m^2^	32 ± 13 mL/m^2^	3	2	1472 (904–2113) pg/mL	N/A	1 (4)
	LBBAP		28 (20–42)	40 (31–44)	81 ± 21 mL/m^2^	50 ± 18 mL/m^2^	3	2	1632 (861–5028) pg/mL	N/A	0
Zizek *et al*. 2022	HBP	6 months	40 (37–45)	46 (41–55)	63.6 (49.6–81) mL/m^2^	32.7 (25.6–42.6) mL/m^2^	3 (3–3)	2 (1.25–2.75)	905 (479–1911) pg/mL	N/A	N/A
Huang *et al*. 2022	HBP	18 months	31.9 ± 7.0	9 months—53.9 ± 11.9 18 months—54.8 ± 9.4	9 months—55.0 ± 9.5 18 months—54.0 ± 5.9	N/A	2.82 ± 0.65	1.50 ± 0.60	IgBNP concentration—2.6 ± 0.6	1 (4.0)	3 (12)
Jin *et al*. 2022	LBBAP	12 months	53.03571429	60.4 ± 8.8	46.3 ± 5.6	N/A	3.0 (2.0–3.3)	2.0 (1.0–2.0)	N/A	2 (4.3)	0
Vijayaraman *et al*. 2022	Combined CSP	27 ± 19 months	46.5 ± 14.2	51.9 ± 11.2	N/A	N/A	N/A	N/A	N/A	43 (39)	CSP—27 (25)
	HBP		46.4 ± 14.1	52.3 ± 10.9	N/A	N/A	N/A	N/A	N/A	N/A	N/A
	LBBAP		46.9 ± 15.1	50.1 ± 12.9	N/A	N/A	N/A	N/A	N/A	N/A	N/A
Chaumont *et al*. 2023	HBP	2.75 ± 1.2 years	Overall—47 ± 14 27.0 ± 8****	HBP—60.0 ± 9.0 52.0 ± 7****	N/A	N/A	3.0 ± 0.7	1.6 ± 0.5	N/A	6 (8)	2 (2.67)
Morina-Vazquez *et al*. 2021	HBP	10.5 (3–12.5) months	Overall—60 (58–63.25) 35 (23.8–45.3)****	HBP—60 (60–65) *P* > 0.05 40 (35–56.5)**** *P* < 0.05	N/A	N/A	20 (56.5)***	1 (2.8)***	N/A	N/A	N/A
Wu *et al*. 2021	Combined CSP	12 months	34.3 ± 7.7	50.6	129.6	70.0	2.85	1.41	250.3 pg/dL Narrow QRS: 251 (134–482) pg/dL LBBB: 248 (108–514) pg/dL	8 (4.7)	CSP—14 (8.2)
	HBP		35.4 ± 7.9	51.1 ± 12.2							N/A
	LBBAP		33.2 ± 7.2	49.7 ± 13.9							N/A
Su *et al*. 2020	HBP	3.0 (2.0–4.4) years	Reduced LVEF group—32.5 ± 5.8% Preserved LVEF group—57.1 ± 10.4	Reduced LVEF group 1 year—49.1 ± 12.9 2 years—49.8 ± 12.2 3 years—49.9 ± 12.6 > 3 years—52.8 ± 14.2 Preserved LVEF group 1 year—62.3 ± 8.1 2 years—63.7 ± 8.1 3 years—63.3 ± 5.1 > 3 years—64.8 ± 6.7	Overall—51.8 ± 8.5 Reduced LVEF—56.2 ± 9.60 Preserved LVEF—47.7 ± 4.50	Reduced LVEF group 1 year—76.8 ± 54.5 2 years—76.4 ± 49.8 3 years—72.1 ± 55.2 > 3 years—59.7 ± 36.5 Preserved LVEF group 1 year—40.3 ± 15 2 years—37.7 ± 20.3 3 years—36.0 ± 11.7 > 3 years—37.3 ± 11.9	Overall 2.78 ± 9.64 Reduced LVEF 3.03 ± 0.64 Preserved LVEF 2.56 ± 0.55	Overall 1.41 ± 0.71 Reduced LVEF 1.57 ± 0.83 Preserved LVEF 1.27 ± 0.55	Overall 220 (134–421) pg/dL Reduced LVEF 198 (131–422) pg/dL Preserved LVEF 241 (137–387) pg/dL	7 (8.64)	14 (17.3)
Sun *et al*. 2020	HBP	3 months	Overall—48.8 ± 11.2 Reduced LVEF—34.4 ± 10	Overall—51.1 ± 9.0 Reduced LVEF—40.0 ± 10.0	N/A	N/A	N/A	N/A	N/A	N/A	N/A
Wang *et al*. 2019	Combined CSP	30.5 months	Overall—34.8 ± 11.2 Reduced LVEF—29.8 ± 5.87	Overall—49.3 ± 14.9 Reduced LVEF—46.2 ± 14.7	N/A	Overall—83.7 ± 62.5 Reduced LVEF—95.8 ± 66.0	2.73 ± 0.59	1.42 ± 0.53	N/A	6 (10.9)	6 (11.5)
Deshmukh *et al*. 2020	HBP + LV pacing	21.1 ± 9.3 months	28.1 ± 8.0	42.6 ± 11.8	135.9 ± 69.7	N/A	N/A	N/A	N/A	0	0
Huang *et al*. 2017	HBP	20 months	Overall—44.9 ± 14.6 Preserved LVEF—56.6 ± 9.9 Reduced LVEF—32.2 ± 4.8	Overall 3 months—56.5 ± 8.7 1 year—59.7 ± 9.8 Last FU—60.0 ± 8.1 Preserved LVEF 3 months—60.1 ± 8.0 1 year—63.2 ± 8.2 Last FU—62.9 ± 6.9 Reduced LVEF 3 months—53.9 ± 8.4 1 year—55.7 ± 10.2 Last FU—57.2 ± 8.7	Overall 3 months—52.7 ± 5.3 1 year—50.6 ± 5.4 Last FU—51.0 ± 5.1 Preserved LVEF 3 months—50.2 ± 4.4 1 year—49.0 ± 4.4 Last FU—49.6 ± 3.9 Reduced LVEF 3 months—54.5 ± 5.3 1 year—52.3 ± 6.0 Last FU—r52.6 ± 5.9	N/A	Preserved LVEF—2.7 ± 0.6 Reduced LVEF—2.9 ± 0.6	Preserved LVEF—1.4 ± 0.5 *P* < 0.001 Reduced LVEF—1.4 ± 0.4 *P* < 0.001	Overall—309.0 ± 254.9 pg/mL Preserved LVEF—308.5 ± 254.1 pg/mL Reduced LVEF—148.3 ± 232.4 pg/mL	2 (4.8)	2 (4.76)
Vijayaraman *et al*. 2017	HBP	19 ± 14 months	Overall—43 ± 13 Reduced LVEF—33 ± 7 Preserved LVEF—56 ± 5	Overall—50 ± 10 Reduced LVEF—45 ± 9 Preserved LVEF—57 ± 7	N/A	N/A	2.50 ± 0.50	1.9 ± 0.5	N/A	3 (7)	5 (11.9)
Occhetta *et al*. 2006	HBP	23 months	52.0 ± 9.1	53.4 ± 7.9	LVEDV—93.2 ± 26.6	LVESV −44.7 ± 17.6	2.33 ± 0.60	1.75 ± 0.4	N/A	N/A	0

HBP, His bundle pacing; LBBAP, left bundle branch associated pacing; CSP, conduction system pacing; N/A, not applicable/available; BVP, biventricular pacing; LVEF, left ventricular ejection fraction; LVEDVI, left ventricular end-diastolic volume index; LVESVI, left ventricular end-systolic volume index; NYHA, New York Heart Association; HF, heart failure; SR, sinus rhythm; NSR, non-sinus rhythm; LVEDD, left ventricular end-diastolic diameter. *LVEDD. **Averaged across both HBP and LBBAP. ***Proportion of patients in the NYHA III-IV category. ****Reduced LV ejection fraction.

### Paced QRS duration

**Figure 4:**
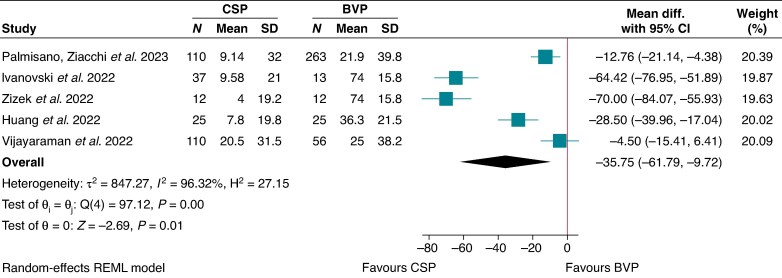
Change in QRS duration - CSP vs. BVP.

The paced QRS duration of CSP was lower in comparison to BVP (MD −35.8, 95% CI −61.8 to −9.72, I^2^ = 96.3; *P* < 0.05)^18,20,30,31,45^ (*Figure 4*).

### Change in NYHA class

Conduction system pacing demonstrated a significant reduction in NYHA class in comparison to BVP (MD −0.53, 95% CI −1.01 to −0.04, I^2^ = 62.1; *P* = 0.03).^30,31,45^ Overall, the rest of the included studies that could not be incorporated in the meta-analysis also demonstrated an improvement in NYHA on F/U.^19,21,22,27,32,33,35,41,43,44^

### Hospitalization

CSP demonstrated a non-significant reduction in hospitalization when also compared to BVP (Log odds ratio −0.49, 95% CI −1.83–0.84, I^2^ = 0.00; *P* = 0.47). From the included studies reporting HF associated hospitalization on F/U, a pooled total of 100 events was observed.^18–22,27–30,33,37,41,43,47^

### Mortality

Similarly, CSP demonstrated a non-significant reduction in mortality when compared to BVP (Log odds ratio −0.80, 95% CI −2.19–0.59, I^2^ = 3.80; *P* = 0.26). Of the included studies reporting outcomes on all-cause mortality, a pooled total of 101 events was observed^19–22,27–33,38,39,41,43,46^ (*Table 7*).

### Follow-up pacing thresholds

The overall increase in pacing thresholds on follow-up was similar for HBP in comparison to BVP (MD −0.03, 95% CI −0.18 to −0.12, I^2^ = 0.00; *P* = 0.70).^18,30,31,45^ Follow-up thresholds for LBBAP were significantly lower in comparison to BVP (MD −0.55, 95% CI −0.72 to −0.39, I^2^ = 0.00; *P* < 0.05).^18,20,31^

### Acute thresholds

His bundle pacing demonstrated a non-significant reduction in the events of acute threshold elevation in comparison to BVP (Log odds ratio −0.69, 95% CI −2.05–0.66, I^2^ = 0.00; *P* = 0.32).^30,31^

### Left ventricular dimensions

Study findings indicated that CSP resulted in a reduction of left ventricle end-systolic and end-diastolic dimensions at follow-up (see Supplementary material online, *Table S5*).^21,28–33,38,41–45^

### Further analysis

**Figure 5:**
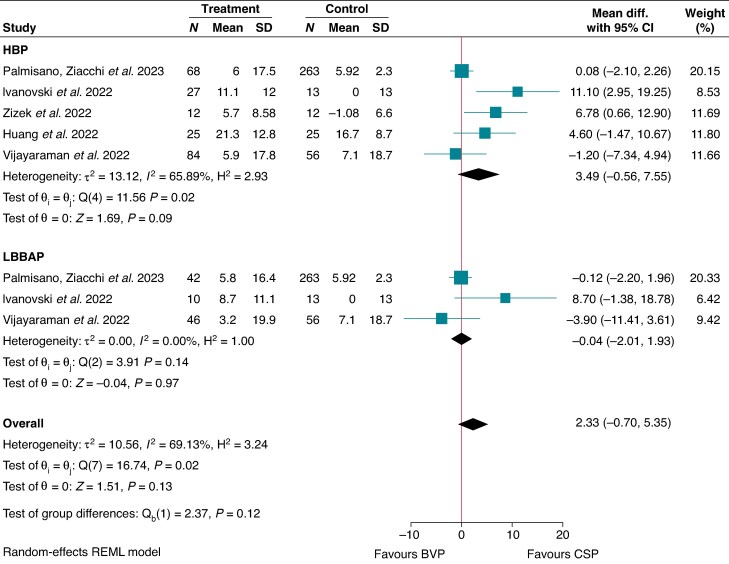
Change in LVEF - Subanalysis - HBP and LBBAP.

His bundle pacing demonstrated a non-significant increase in LVEF compared to BVP by 3.49% (MD 3.49, 95% CI −0.56–7.55; I^2^ = 65.9%; *P* = 0.09),^18,20,30,31,45^ while LBBAP demonstrated similar outcomes to BVP (MD −0.04, 95% CI −2.01–1.93; I^2^ = 0.00; *P* = 0.97)^18,20,31^ (*Figure 5*). Subgrouping according to mean baseline LVEF revealed a non-significant improvement in LVEF for CSP in comparison to BVP [LVEF < 40: (MD 7.07, 95% CI 1.37–12.8; I^2^ = 28.7%, *P* = 0.24); LVEF > 40: (MD 1.13, 95% CI −3.02–5.27; I^2^ = 65.5, *P* = 0.08)]. His bundle pacing exhibited similar outcomes of LVEF with LBBAP (MD 1.65, 95% CI −2.45–5.76; I^2^ = 0.00%; *P* = 0.86).^18,20,31^

The HBP group demonstrated a greater reduction in paced QRS duration compared to BVP (MD −35.2, 95% CI −63.7 to −6.76, I^2^ = 96.5; *P* < 0.05).^18,20,30,31,45^ Left bundle branch area pacing also demonstrated a greater reduction when compared to BVP (MD −27.4, 95% CI −52.5 to −2.17, I^2^ = 90.1; *P* < 0.05).^18,20,31^ There was minimal difference in paced QRS duration outcomes between HBP and LBBAP (MD 1.70, 95% CI −16.6–20.0, I^2^ = 83.5; *P* = 0.86).^18,20,31^

Analysis revealed a statistically significant improvement in NYHA class for HBP in comparison to BVP (MD −0.55, 95% CI −1.06 to −0.04, I^2^ = 66.5; *P* = 0.04).^30,31,45^ Analysis could not be performed for the LBBAP intervention as only one included study reported NYHA outcomes for the LBBAP cohort.

Analysis revealed a significant reduction in elevated thresholds for LBBAP in comparison to BVP (MD −0.55, 95% CI −0.72 to −0.39, I^2^ = 0.00; *P* < 0.05). His bundle pacing did not demonstrate any significant differences to BVP (MD −0.03, 95% CI −0.18–0.12, I^2^ = 0.00; *P* = 0.70).

Sensitivity analysis according to study methodology was performed with included studies divided in to RCTs and observational studies. There was a significant difference in ΔNYHA functional class between the two methodologies in favour of CSP [observational studies: MD (−)0.78 (95% CI −1.16 to −0.40, I^2^ = 0.00%, *P* < 0.05) vs. RCT: MD (−)0.08 (95% CI −0.55–0.39, *P* = 0.74), Q_b_(1) = 5.20, *P* = 0.02]. There were no significant differences between methodologies for ΔLVEF (Q_b_(1) = 0.11, *P* = 0.74), ΔQRSd (Q_b_(1) = 0.26, *P* = 0.61), ΔHFH (Q_b_(1) = 0.05, *P* = 0.82), value of acute thresholds (Q_b_(1) = 0.64, *P* = 0.42), and thresholds on follow-up (Q_b_(1) = 0.04, *P* = 0.85).

### Meta-regression

To explore potential sources of heterogeneity, meta-regression was applied to the test the influence of mean age, male proportion, baseline LVEF, and baseline QRSd (see Supplementary material online, *Table S6*). Baseline QRSd contributed significantly to heterogeneity for change in LVEF (*P* = 0.0490) and change in QRSd (*P* = 0.0470). Mean age and male proportion contributed significantly to heterogeneity for change in NYHA.

### Quality assessment and publication bias

The outcomes of the risk of bias assessment for each study are shown in *Figure 2*. Of the randomized studies, one study demonstrated some concerns of bias.^30^ Of the non-randomized studies, 2 studies demonstrated low risk of bias,^43,47^ 13 studies demonstrated some concerns,^18,19,22,27–29,32,33,35,37,40–42^ and 9 studies exhibited high risk of bias.^20,21,31,34,36,38,39,44,45^

The funnel plot for change in LVEF, QRSd, and NYHA class for CSP vs. BVP showed an asymmetrical distribution of similar weight studies (see Supplementary material online, *Figure S1*). The Egger regression test was performed and revealed evidence of significant publication bias (*P* = 0.0115) for QRSd; however, no evidence of significant bias was observed for change in LVEF (*P* = 0.119) and NYHA (*P* = 0.619).

## Discussion

The findings from this systematic review and meta-analysis demonstrate that CSP can be implemented successfully with AVNA and may result in improvements in LV function and dimensions compared to conventional BVP. The extent of QRS narrowing, as a measure of electrical synchronization, was more favourable after CSP implantation. Furthermore, patients that underwent CSP demonstrated a trend of better clinical outcomes in the context of NYHA classification along with reduced HFH in comparison to BVP. With respect to periprocedural outcomes, there were no significant differences observed in procedural and fluoroscopy times. Additionally, CSP demonstrated a similar periprocedural complication and subacute adverse event rate. Certain findings were consistent with the meta-analysis performed by Xu *et al.*^23^ although there were also notable differences in outcomes. Xu *et al.*^23^ reported a significant improvement in LVEF and QRSd for HBP, however, in combination with a lower procedural success rate in addition to more complications. This contrasted with this review’s findings where 95.9% of HBP procedures were successfully facilitated and complication rates were significantly lower. This might be due to the fact that we included more studies and patients than the previous study. While further clinical trials of CSP with AVNA are awaited, this review highlights notable implications for clinical practice and future research.

The application of AVNA with permanent pacing strategies has emerged as an effective therapeutic approach for improving symptoms and quality of life in patients experiencing AF with rapid ventricular rates refractory to other treatment modalities.^48^ AHA/ACC/HRS Atrial Fibrillation Practise Guidelines (2014) recommend consideration of AVNA with permanent RVP in patients exhibiting AF with high ventricular rates when pharmacological therapy is suboptimal and rhythm control cannot be attained (class IIa, level of evidence B).^7^ While effective rate control derived from AVNA significantly addresses symptoms, the chronic deleterious effects of RVP in patients with impaired LV function have encouraged the search for alternative pacing modalities. The use of BVP with AVNA was associated with a reduction in heart failure associated mortality and HFH along with an improvement in QOL and LVEF, when compared with RVP.^48,49^ Delayed left ventricular activation with a present left bundle branch block may result in unfavourable physiology in patients with HF. While long-term studies have demonstrated the reduction in morbidity and mortality through BVP, implant of the left ventricular lead is not always feasibility and in certain patient populations, BVP may only provide modest correct in QRS and ventricular activation time.^17,50,51^ as well as a recognized non-response rate to BVP in CRT patients. As a result, CSP has emerged as a potential alternative with Deshmukh *et al*.^52^ first reporting feasibility for the HBP approach in 2000. Further studies have followed on and demonstrated feasibility and efficacy in improving echocardiographic outcomes and functional capacity.^53–55^ Sharma *et al.*^56^ demonstrated the effectiveness of HBP as a rescue approach for patients exhibiting failed coronary sinus lead implantation or suboptimal/non-response to BVP. In spite of the modest crossover rates from HBP to BVP, Vinther *et al.*^57^ reported a significant increase in LVEF with CSP in the per-protocol analysis. In consideration of HBP limitations, Huang *et al.* proposed the LBBAP approach as it presented certain technical advantages including lower thresholds, application for more distal diseases, and a faster learning curve. An RCT conducted by Vijayaraman *et al.*^58^ demonstrated a significant increase in LVEF when patients were randomized to either HBP or LBBAP instead of BVP. In spite of the technical advantages LBBAP confers, a study published by Ali *et al.*^59^ published findings suggesting a lesser degree of physiological ventricular activation. Several meta-analysis have also reinforced the consensus from the observational studies and controlled trials by demonstrating findings of superior echocardiographic and functionality outcomes in comparison to BVP.^9,60–62^

Recent observational studies have demonstrated findings suggestive of feasibility and efficacy for HBP and LBBAP when performed in conjunction with AVNA.^19,21,31,41^ These studies have demonstrated that CSP with AVNA is associated with improved echocardiographic outcomes, maintenance of physiologic ventricular activation, and functional outcomes. The findings from this meta-analysis have potentially reinforced this hypothesis as non-significant improvements in LVEF along with QRS narrowing in comparison to BVP were evident. While the improvement in LVEF was not statistically significant, it is important to note that there was heterogeneity in patient allocation to the respective treatment groups. The study conducted by Vijayaram *et al.* 2022 was the only included study to demonstrate a superior improvement in LVEF for BVP in comparison to CSP; however, the authors reported a significant discrepancy in baseline LVEF between the BVP and CSP cohorts.^20^ The BVP cohort exhibited a baseline LVEF of 26.7 ± 10.5% in a significant contrast to the CSP cohort that exhibited an LVEF of 46.5 ± 14.2%. This would likely affect the degree of possible improvement in LVEF and thus the comparisons between the two cohorts may not truly be reflective of their real efficacy. The exclusion of the study from the meta-analysis resulted in a mean difference of 4.57% (95% CI −0.06–9.20, I^2^ = 69.0, *P* = 0.05) for improvement in LVEF between CSP and BVP.

The improvement in LVEF was even more pronounce in patients with severely impaired LVEF at baseline although interestingly the association of baseline LVEF with post-procedural LVEF was not significant on meta-regression. Baseline QRSd was noted as a significant source of heterogeneity for change in LVEF and QRSd as per meta-regression.

Atrioventricular node ablation can be potentially challenging especially in patients with HBP as it evokes a risk of increasing HBP thresholds when ablating near to the HBP lead.^19^ This systematic review observed a pooled prevalence of 34 cases with HBP and AVNA (3.22%) demonstrating acute elevation of thresholds with six cases requiring lead repositioning. Left bundle branch area pacing presents as a reliable alternative to attaining low and stable pacing thresholds, as made evident by no patients in the pooled LBBAP that sustained acute elevations in thresholds. Within the studies also including a BVP control population, three cases of acute threshold elevation were observed with one lead revision required for BVP from a total of 369 patients. In view of the concerns surrounding acute thresholds, the 2021 European Society of Cardiology (ESC) pacing guidelines recommend implantation of a backup ventricular lead in patients with HBP undergoing AVNA that is evident with the significant pooled prevalence of backup RV lead insertion in this systematic review.^63^ A majority of AVNA was safely performed during the CSP implantation procedure through femoral or axillary approach. The remaining cases had AVNA generally performed within 2–6 weeks with operators potentially preferring ablation in subacute setting once thresholds were confirmed to be stable. Within the studies including a conventional pacing (CP) control population, the pooled incidence of acute periprocedural adverse events not including acute threshold elevation was similar between CSP (0.529%) and CP (0.901%). These pooled observations support the application of CSP as a safe and acceptable alternative approach in patients receiving AVNA compared to CP. Additionally, the findings were consistent with the reported outcomes from the Multicentre European Left Bundle Branch Area Pacing Outcomes Study (MELOS) that also reported similar rates between the two approaches.^64^ There was a greater incidence of recurrence of AV node conduction in the CSP group (3.88%) compared to the CP group (1.41%). This could be explained by a reluctance to administering additional ablation for lesions for concern of increasing the HBP thresholds. The safety and efficacy of CSP with AVNA were further supported by the reduction in all-cause mortality and HFH on follow-up along with stable thresholds in comparison to BVP.

Given the necessity for further prospective and comparative studies, several clinical trials evaluating the efficacy of CSP with AVNA are ongoing (CONDUCT-AF Trial, RAFT-P&A, PACE-FIB, LBBAP-AFHF).^65–68^ Additionally, there is interest in facilitating trials with multiple arms including pulmonary vein isolation vs. AVNA with CSP in order to compare and guide clinical practice for treatment strategies of AF. Regarding patient selection, a greater mechanistic understanding of which factors associated with AF are more detrimental to myocardial function, at both a cellular and organ level, are essential. Furthermore, the capacity to determine which patients exhibit a propensity for arrhythmia-induced cardiomyopathy will be highly efficacious as HF is not ubiquitous to all patients with AF. Overall, the promising preliminary findings suggest CSP’s potential to contribute to the armamentarium for AF through its ability to approximate physiological activation of the ventricles although further methodologically robust longitudinal studies are required to assess long-term safety and effectiveness.

### Study limitations

This systematic review and meta-analysis exhibited numerous limitations. Several studies utilized small samples sizes along with significant differences in methodologies. Some of the studies retrieved were of lower evidence with overall limited prospective and randomized controlled trials published for this area. This was not unexpected given the relative infancy of CSP in pace and ablate strategies. While meta-analysis would ideally be conducted between studies of the same methodologies, the paucity of RCTs led to the inclusion of observational studies. As a result, sensitivity analysis was performed to examine the effect of the methodologies on the overall outcomes. Majority of the outcomes remained consistent between the methodologies aside from the change in NYHA functional classification in favour of CSP. Overall, in view of the increasing dissemination of this approach, we can anticipate the facilitation of more robust studies and trials in the future. The sample size across the included studies was generally lower with seldom use of statistical power calculation. Several studies did not facilitate a comparator arm and thus it was challenging to compare the efficacy and safety of CSP with BVP for AVNA. Moderate to severe heterogeneity was observed for majority of the outcomes evaluated with meta-analysis. This was potentially due to significant crossover between CSP therapies in the included studies as further analysis appeared to have isolated heterogeneity to either approach (HBP or LBBAP). Additionally, there was variability in reported demographics and outcomes across studies and thus further evaluation of heterogeneity through subgroup analysis and meta-regression proved challenging. Majority of studies exhibited a short follow-up period of ∼6–12 months and thus, the long-term outcomes are still to be determined. Furthermore, most of the studies were single-centre and thus the external validity of results could have been compromised. Lastly, there was significant variation in the reporting of certain outcomes such as periprocedural adverse events.

## Conclusion

In conclusion, the findings from our systematic review and meta-analysis conveyed superior electrocardiographic, echocardiographic, and clinical outcomes regarding functional status, and non-significant improvement in mortality and HHF for CSP in comparison to BVP. As a result, the outcomes of this meta-analysis suggest that CSP is promising alternative to BVP for facilitating pace and ablation strategies with AVNA for patients with AF. However, large scale robust controlled trials with appropriate longitudinal follow-up are required to validate the efficacy of CSP as an alternative approach to BVP in conjunction with AVNA.

## Supplementary Material

euaf106_Supplementary_Data

## Data Availability

Data are available on request.
