# Supplementary material for: A comparative analysis of conduction system pacing and biventricular pacing in patients undergoing atrioventricular node ablation: a systematic review and meta-analysis
Source: Europace. 2025 Jul 11;27(7):euaf106. doi: 10.1093/europace/euaf106 (PMC12255165; doi:10.1093/europace/euaf106)
Supplement: euaf106_Supplementary_Data [file euaf106_supplementary_data.zip › Supplement Caption.docx]

**Caption:** Funnel Plot Assessment
