# Supplementary material for: A comparative analysis of conduction system pacing and biventricular pacing in patients undergoing atrioventricular node ablation: a systematic review and meta-analysis
Source: Europace. 2025 Jul 11;27(7):euaf106. doi: 10.1093/europace/euaf106 (PMC12255165; doi:10.1093/europace/euaf106)
Supplement: euaf106_Supplementary_Data [file euaf106_supplementary_data.zip › Supplementary Tables.docx]

| **Supplementary Table 1. Conventional population demographics** | | | | | | | | | |  |  |  |  |  |
| --- | --- | --- | --- | --- | --- | --- | --- | --- | --- | --- | --- | --- | --- | --- |
| **Study** | **Specified intervention** | **Age** | **Gender-Male Prevalence** | **Body Mass Index** | **Co-morbidities** | | | | | | | **AF Classification** | | |
|  |  |  |  |  | **CAD** | **DM** | **HTN** | **CVA** | **CKD** | | **COPD** | **PAF** | **Persistent AF** | **Permanent AF** |
| Palmisano, Parlavecchio, Crea et al. 2023 | N/A | 79.0 | 31.1 | *Obesity prevalence - 21.0 | N/A | N/A | N/A | N/A | N/A | | N/A | N/A | N/A | N/A |
| Ivanovski et al. 2023 | N/A | 71 +/- 8 | 42.6 | N/A | N/A | N/A | N/A | N/A | N/A | | N/A | N/A | N/A | N/A |
| Liu et al. 2023 | N/A | 78 +/- 5 | 37% | N/A | N/A | N/A | N/A | N/A | N/A | | N/A | N/A | N/A | N/A |
| Palmisano, Parlaveccio, Vetta et al. 2023 | N/A | 79.0 +/- 9.1 | 33.6 | N/A | N/A | N/A | N/A | N/A | N/A | | N/A | N/A | N/A | N/A |
| Palmisano, Ziacchi et al. 2023 | BVP | 75.3 +/- 8.7 | 53.9 | *Obesity prevalence - 27.1 | 115 (43.7) | 98 (37.3) | 206 (78.3) | 35 (13.3) | 104 (39.5) | | 71 (27) | 0 | 0 | 263 (100) |
| Rijks et al. 2023 | N/A | 79.2 +/- 4.2 | 40% | 27.0 +/- 4.6 | N/A | N/A | N/A | N/A | N/A | | N/A | N/A | N/A | N/A |
| Qi et al. 2023 | N/A | 74.6 +/- 7.1 | 29.0 | N/A | N/A | N/A | N/A | N/A | N/A | | N/A | N/A | N/A | N/A |
| Nam et al. 2023 | N/A | 72 +/- 10 | 4 (50) | N/A | N/A | N/A | N/A | N/A | N/A | | N/A | N/A | N/A | N/A |
| Ye et al. 2023 | N/A | 68.5 +/- 7.7 | 61 | N/A | N/A | N/A | N/A | N/A | N/A | | N/A | N/A | N/A | N/A |
| Cai et al. 2023 | N/A | 69.7 +/- 9.7 | 48.5 | N/A | N/A | N/A | N/A | N/A | N/A | | N/A | N/A | N/A | N/A |
| Pillai et al. 2023 | N/A | 76.4 | 55 (56.1) | N/A | N/A | N/A | N/A | N/A | N/A | | N/A | N/A | N/A | N/A |
| Ivanovski et al. 2022 | BVP | 70 (67-73.5) | 53.8 | N/A | 6 (46.2) | 3 (23.1) | 9 (69.2) | N/A | N/A | | N/A | N/A | N/A | N/A |
| Zizek et al. 2022 | BVP | 69.3 +/- 6.6 | 58.3 | N/A | 6 (50) | 2 (16.7) | 9 (75) | N/A | N/A | | N/A | N/A | N/A | 12 (100) |
| Huang et al. 2022 | BVP | 65.0 +/- 8.2 | 68 | N/A | 6 (24) | 4 (8) | 15 (60) | N/A | Creatinine - 1.1 +/- 0.3 mg/dl | | N/A | N/A | 25 (100) | N/A |
| Jin et al. 2022 | N/A | 75.3 | 30 (53.6) | 24.3 | N/A | N/A | N/A | N/A | N/A | | N/A | N/A | N/A | N/A |
| Vijayaraman et al. 2022 | N/A | 75 +/- 10 | 54 (48) | 30 +/- 6 | 42 (37) | 24 (21) | 65 (57) | N/A | N/A | | N/A | 27 (24) | 64 (57) | 22 (20) |
| Chaumont et al. 2023 | N/A | 71 +/- 12 | 42 (51) | 28 +/- 6 | N/A | N/A | N/A | N/A | N/A | | N/A | N/A | N/A | N/A |
| Morina-Vazquez et al. 2021 | N/A | 77 (70-81) | 11 (28.2) | N/A | N/A | N/A | N/A | N/A | N/A | | N/A | N/A | N/A | N/A |
| Wu et al. 2021 | N/A | 69.3 +/- 10.1 | 103 (60.6) | 24 +/- 3.2 | N/A | N/A | N/A | N/A | N/A | | N/A | N/A | N/A | N/A |
| Su et al. 2020 | N/A | 70.1 +/- 10.5 | 57.4 | *24.4 +/- 3.8 | N/A | N/A | N/A | N/A | N/A | | N/A | N/A | N/A | N/A |
| Sun et al. 2020 | N/A | 72.2 +/- 8.7 | 9 (52.9) | N/A | N/A | N/A | N/A | N/A | N/A | | N/A | N/A | N/A | N/A |
| Wang et al. 2019 | N/A | 67.6 +/- 10.9 | 37 (71.2) | N/A | N/A | N/A | N/A | N/A | N/A | | N/A | N/A | N/A | N/A |
| Deshmukh et al. 2020 | N/A | 69.9 +/- 10.7 | 10 (76.9) | N/A | N/A | N/A | N/A | N/A | N/A | | N/A | N/A | N/A | N/A |
| Huang et al. 2017 | N/A | 72.8 +/- 8.3 | 26 (61.9) | N/A | N/A | N/A | N/A | N/A | N/A | | N/A | N/A | N/A | N/A |
| Vijayaraman et al. 2017 | N/A | 74 +/- 11 | 19 (45) | N/A | N/A | N/A | N/A | N/A | N/A | | N/A | N/A | N/A | N/A |
| Occhetta et al. 2006 | RVP | 71.4 +/- 5.6 | 9 (50) | N/A | 5 (27.8) | N/A | 7 (38.9) | N/A | N/A | | N/A | N/A | N/A | N/A |
| CAD *Coronary artery disease;* DM *Diabetes mellitus;* HTN *Hypertension;* CVA *Cerebrovascular accident;* CKD *Chronic kidney disease;* COPD *Chronic obstructive pulmonary disease;* AF *Atrial fibrillation;* PAF *Paroxysmal atrial fibrillation;* BVP *Biventricular pacing;* RVP *Right ventricular pacing,* N/A *Not applicable/available* | | | | | | | | | | | | | | |

| **Supplementary Table 2. Medications at baseline** | | | | | | |
| --- | --- | --- | --- | --- | --- | --- |
| **Study** | **Specified intervention** | **Medication** | | | | |
|  |  | **Beta-blockers (%)** | **CCB (%)** | **Digoxin (%)** | **Antiarrhythmics (%)** | **ARB/ACEI/ARNI(%)** |
| Palmisano, Parlavecchio, Crea et al. 2023 | Combined CSP | N/A | N/A | N/A | N/A | N/A |
| Ivanovski et al. 2023 | Combined CSP | 64 (94.1) | N/A | 17 (25) | 17 (25) | 42 (61.8) |
| Liu et al. 2023 | LBBAP | 20 (83) | 10 (42) | 5 (21) | 12 (50) - Class I - 3 (13), Class III - 9 (38) | N/A |
| Palmisano, Parlaveccio, Vetta et al. 2023 | Combined CSP | 102 (95.3) | 13 (12.1) | 27 (25.2) | 9 (8.4) | 40 (37.4) |
| Palmisano, Ziacchi et al. 2023 | HBP | 58 (85.3) | 8 (11.8) | 18 (26.5) | Amiodarone - 7 (10.3), dronedarone 1 (1.5), overall, 8 (11.8) | 45 (66.2) |
|  | LBBAP | 40 (95.2) | 5 (11.9) | 11 (26.2) | Amiodarone - 3 (7.1), dronedarone - 0 (0) - overall 3 (7.1) | 26 (61.9) |
| Rijks et al. 2023 | LBBAP | 13 (52) | 11 (44) - DHP 0 5 (20), non-DHP - 6 (24) | 5 (20) | 12 (48) - Flecainide - 2 (8), sotalol - 5 (20), amiodarone - 5 (20) | 14 (56) |
| Qi et al. 2023 | Combined CSP | 26 (83.9) | 5 (16.1) | 11 (35.5) | Amiodarone - 3 (9.7) | N/A |
| Nam et al. 2023 | Combined CSP | N/A | N/A | N/A | N/A | N/A |
| Ye et al. 2023 | Combined CSP | 9 (47.4) | N/A | N/A | N/A | 1 (5.3) |
| Cai et al. 2023 | LBBAP | 73 (84.9) | N/A | 18 (20.9) | N/A | 64 (74.4) |
|  | HBP | 76 (88.4) | N/A | 16 (18.6) | N/A | 64 (74.4) |
| Pillai et al. 2023 | HBP | N/A | N/A | N/A | N/A | N/A |
|  | LBBAP | N/A | N/A | N/A | N/A | N/A |
| Ivanovski et al. 2022 | HBP | 25 (92.6) | N/A | 7 (25.9) | Amiodarone - 6 (22.2) | 20 (74.1) |
|  | LBBAP | 9 (90) | N/A | 2 (20) | Amiodarone - 1 (10) | 6 (60) |
| Zizek et al. 2022 | HBP | 12 (100) | N/A | 3 (25) | Amiodarone - 2 (16.7) | 9 (75) |
| Huang et al. 2022 | HBP | N/A | N/A | N/A | N/A | N/A |
| Jin et al. 2022 | LBBAP | 31 (55.4) | N/A | 16 (25) | N/A | 32 (57.1) |
| Vijayaraman et al. 2022 | Combined CSP | 103 (94) | 15 (14) | 26 (24) | 76 (69) | 56 (51) |
| Chaumont et al. 2023 | HBP | N/A | N/A | N/A | N/A | N/A |
| Morina-Vazquez et al. 2021 | HBP | N/A | N/A | N/A | N/A | N/A |
| Wu et al. 2021 | Combined CSP | 147 (86.5) | N/A | 49 (28.8) | N/A | 135 (79.4) |
| Su et al. 2020 | HBP | * 73 (83.6) | N/A | * 22 (28.2) | N/A | * 55 (70.5) |
| Sun et al. 2020 | HBP | N/A | N/A | N/A | N/A | N/A |
| Wang et al. 2019 | Combined CSP | 42 (80.8) | 5 (9.6) | 24 (46.2) | Amiodarone - 13 (25.0) | 44 (84.6) |
| Deshmukh et al. 2020 | HBP + LV pacing | 9 (69) | N/A | N/A | N/A | 11 (85) |
| Huang et al. 2017 | HBP | 40 (95.2) | N/A | 20 (47.6) | N/A | 36 (85.7) |
| Vijayaraman et al. 2017 | HBP | N/A | N/A | N/A | N/A | N/A |
| Occhetta et al. 2006 | HBP | N/A | N/A | N/A | N/A | N/A |
| CCB *Calcium channel blocker;* ARB *Angiotensin receptor blocker;* ACEi *Angiotensin converting enzyme inhibitor;* ARNI *Angiotensin receptor-neprilysin inhibitor;* CSP *Conduction system pacing;* HBP *His Bundle pacing;* LBBAP *Left bundle branch area pacing;* DHP *Dihydropyridine, LV* Left ventricular | | | | | | |

| **Supplementary Table 3. Biventricular pacing periprocedural details** | | | | | | | | | | | |
| --- | --- | --- | --- | --- | --- | --- | --- | --- | --- | --- | --- |
| **Study** | **Specified intervention** | **Successful implantation – BVP (%)** | **Implantation procedural time/min** | **Fluoroscopy duration/min** | **Type of device implanted** | | | | | **Back-up RV lead (%)** | **Atrial lead implantation (%)** |
|  |  |  |  |  | **Dual chamber PPM (%)** | **Biventricular PPM (%)** | **Dual chamber ICD** | | **Biventricular ICD** |  |  |
| Palmisano, Parlavecchio, Crea et al. 2023 | N/A | N/A | N/A | N/A | N/A | N/A |  | N/A | N/A | N/A | N/A |
| Ivanovski et al. 2023 | N/A | N/A | N/A | N/A | N/A | N/A |  | N/A | N/A | N/A | N/A |
| Liu et al. 2023 | N/A | N/A | N/A | N/A | N/A | N/A |  | N/A | N/A | N/A | N/A |
| Palmisano, Parlaveccio, Vetta et al. 2023 | N/A | N/A | N/A | N/A | N/A | N/A |  | N/A | N/A | N/A | N/A |
| Palmisano, Ziacchi et al. 2023 | BVP | 263 (100) | 103.9 +/- 65.7 | 23.5 +/- 28.5 | 0 | 172 (65.4) |  | 0 (0) | 91 (34.6) | N/A | 98 (37.3) |
| Rijks et al. 2023 | N/A | N/A | N/A | N/A | N/A | N/A |  | N/A | N/A | N/A | N/A |
| Qi et al. 2023 | N/A | N/A | N/A | N/A | N/A | N/A |  | N/A | N/A | N/A | N/A |
| Nam et al. 2023 | N/A | N/A | N/A | N/A | N/A | N/A |  | N/A | N/A | N/A | N/A |
| Ye et al. 2023 | N/A | N/A | N/A | N/A | N/A | N/A |  | N/A | N/A | N/A | N/A |
| Cai et al. 2023 | N/A | N/A | N/A | N/A | N/A | N/A |  | N/A | N/A | N/A | N/A |
| Pillai et al. 2023 | N/A | N/A | N/A | N/A | N/A | N/A |  | N/A | N/A | N/A | N/A |
| Ivanovski et al. 2022 | BVP | 13 (100) | N/A | 14 (11-21.5) | 0 | 10 (76.9) |  | 0 | 3 (23.1) | 13 (100) | 13 (100) |
| Zizek et al. 2022 | BVP | 12 (100) | N/A | 16.8 +/- 7.6 | 0 | 9 (75) |  | 0 | 3 (25) | 12 (100) | 12 (100) |
| Huang et al. 2022 | BVP | 50 (100) | N/A | N/A | 0 | 20 (40) |  | 0 | 30 (60) | 50 (100) | N/A |
| Jin et al. 2022 | N/A | N/A | N/A | N/A | N/A | N/A |  | N/A | N/A | N/A | N/A |
| Vijayaraman et al. 2022 | CV (RVP or BVP) | 56 (50) | N/A | 16 +/- 15 | 34 (30) * Dual chamber - both PPM and ICD | 58 (51) * Biventricular device - both PPM and ICD |  | 34 (30) * Dual chamber - both PPM and ICD | 58 (51) * Biventricular device - both PPM and ICD | 113 (100) | 113 (100) |
| Chaumont et al. 2023 | N/A | N/A | N/A | N/A | N/A | N/A |  | N/A | N/A | N/A | N/A |
| Morina-Vazquez et al. 2021 | N/A | N/A | N/A | N/A | N/A | N/A |  | N/A | N/A | N/A | N/A |
| Wu et al. 2021 | N/A | N/A | N/A | N/A | N/A | N/A |  | N/A | N/A | N/A | N/A |
| Su et al. 2020 | N/A | N/A | N/A | N/A | N/A | N/A |  | N/A | N/A | N/A | N/A |
| Sun et al. 2020 | N/A | N/A | N/A | N/A | N/A | N/A |  | N/A | N/A | N/A | N/A |
| Wang et al. 2019 | N/A | N/A | N/A | N/A | N/A | N/A |  | N/A | N/A | N/A | N/A |
| Deshmukh et al. 2020 | N/A | N/A | N/A | N/A | N/A | N/A |  | N/A | N/A | N/A | N/A |
| Huang et al. 2017 | N/A | N/A | N/A | N/A | N/A | N/A |  | N/A | N/A | N/A | N/A |
| Vijayaraman et al. 2017 | N/A | N/A | N/A | N/A | N/A | N/A |  | N/A | N/A | N/A | N/A |
| Occhetta et al. 2006 | RVP | N/A | N/A | *18 +/- 19 | 18 (100) | N/A |  | N/A | N/A | N/A | N/A |
| BVP *Biventricular pacing;* PPM *Permanent pacemaker;* ICD *Implantable cardioverter defibrillator;* RV *Right ventricle;* N/A *Not applicable/available;* | | | | | | | | | | | |

| **Supplementary Table 4. Echocardiographic outcomes – Left ventricular ejection fraction** | | | | | | | | | | |
| --- | --- | --- | --- | --- | --- | --- | --- | --- | --- | --- |
| **Study** | **Conduction System Pacing – LVEF Outcomes** | | | | | **Biventricular Pacing – LVEF Outcomes** | | | | |
|  | **Specified intervention** | **Baseline LVEF (%)** | **F/U – LVEF%** | **Change in LVEF – P value** | **Calculated change in LVEF** | **Specified intervention** | **Baseline LVEF (%)** | **F/U – LVEF%** | **Change in LVEF – P value** | **Calculated change in LVEF** |
| Palmisano, Parlavecchio, Crea et al. 2023 | CSP - Overall | 44.8 ± 11.8 | 49.9 ± 7.6 | <0.001 | 5.1 ± 14 | N/A | N/A | N/A | N/A | N/A |
| Ivanovski et al. 2023 | CSP - Overall | 40 ± 15 | 50.2 | N/A | 10.2 | N/A | N/A | N/A | N/A | N/A |
|  | CSP - SR | 40± 15 | 52 ± 8 | 0.174 | 12 ± 17 | N/A | N/A | N/A | N/A | N/A |
|  | CSP - NSR | 40 ± 16 | 50 ± 14 | <0.001 | 10 ± 21.3 | N/A | N/A | N/A | N/A | N/A |
| Liu et al. 2023 | LBBAP | 44.8 ± 11.8 | 46 ± 12 | >0.05 | 1.2 ± 16.8 | N/A | N/A | N/A | N/A | N/A |
| Palmisano, Parlaveccio, Vetta et al. 2023 | CSP - Overall | 44.8 ± 11.8 | 49.9 ± 7.6 | <0.001 | 5.1 ± 14 | N/A | N/A | N/A | N/A | N/A |
| Palmisano, Ziacchi et al. 2023 | HBP | 40.8 ± 12.0 | 46.8 ± 12.8 | 0.006 | 6 ± 17.5 | BVP | 41.7 ± 10.4 | 47.6 ± 11.1 | <0.001 | 5.9 ± 2.3 |
|  | LBBAP | 42.6 ± 10.9 | 48.4 ± 12.3 | 0.025 | 5.8 ± 16.4 | N/A | N/A | N/A | N/A | N/A |
| Rijks et al. 2023 | LBBAP | 53 ± 7 | N/A | N/A | N/A | N/A | N/A | N/A | N/A | N/A |
| Qi et al. 2023 | CSP - Overall | 60.6 ± 12.1 | 63.7 ± 5.8 | 0.12 | 3.1 ± 13.4 | N/A | N/A | N/A | N/A | N/A |
| Nam et al. 2023 | CSP - Overall | 53.0 ± 4.00 | N/A | N/A | N/A | N/A | N/A | N/A | N/A | N/A |
| Ye et al. 2023 | HBP | 36.56 ± 4.94 | 52.6 ± 5.24 | <0.001 | 16.04 ± 7.2 | N/A | N/A | N/A | N/A | N/A |
|  | LBBAP | 36.56 ± 4.94 | 57.3 ± 11.2 | 0.002 | 20.74 ± 12.2 | N/A | N/A | N/A | N/A | N/A |
| Cai et al. 2023 | LBBAP - Year 1 | 40.7 ± 14.8 | 52.7 ± 14.7 | <0.001 | 12 ± 20.9 | N/A | N/A | N/A | N/A | N/A |
|  | LBBAP - Year 2 | 40.7 ± 14.8 | 54.6 ± 14.8 | <0.001 | 13.9 ± 20.9 | N/A | N/A | N/A | N/A | N/A |
|  | LBBAP - Year 3 | 40.8 ± 14.8 | 57.1 ± 13.9 | <0.001 | 16.3 ± 20.3 | N/A | N/A | N/A | N/A | N/A |
|  | HBP - Year 1 | 41.6 ± 14.3 | 54.9 ± 12.8 | <0.001 | 13.3 ± 19.2 | N/A | N/A | N/A | N/A | N/A |
|  | HBP - Year 2 | 39.8 ± 13.1 | 54.7 ± 13.3 | <0.001 | 14.9 ± 18.7 | N/A | N/A | N/A | N/A | N/A |
|  | HBP - Year 3 | 42.1 ± 16.4 | 58.0 ± 11.4 | <0.001 | 15.9 ± 20 | N/A | N/A | N/A | N/A | N/A |
| Pillai et al. 2023 | CSP - Overall | 53.0 ± 9.7 | 55.0 ± 9.60 | 0.09 | 2 ± 13.6 | N/A | N/A | N/A | N/A | N/A |
|  | CSP - Reduced LVEF | 38.0 ± 7.6 | 46.0 ± 13.3 | 0.02 | 8 ± 15.3 | N/A | N/A | N/A | N/A | N/A |
|  | HBP - Overall | 53 | 55 | 0.12 | 2 | N/A | N/A | N/A | N/A | N/A |
|  | HBP - Reduced LVEF | 38 | 47 | 0.1 | 9 | N/A | N/A | N/A | N/A | N/A |
|  | LBBAP - Overall | 53 | 54 | 0.45 | 1 | N/A | N/A | N/A | N/A | N/A |
|  | LBBAP - Reduced LVEF | 38 | 45 | 0.17 | 7 | N/A | N/A | N/A | N/A | N/A |
| Ivanovski et al. 2022 | HBP | 39 (30-45) | 49 (42-58) | <0.001 | 10 | BVP | 38 (35-40) | 37 (35-41) | 0.916 | -1 |
|  | LBBAP | 28 (20-42) | 40 (31-44) | 0.041 | 12 | N/A | N/A | N/A | N/A | N/A |
| Zizek et al. 2022 | HBP | 40 (37-45) | 46 (41-55) | 0.01 | 6 | 39 (35-40) | 38 (35-42) | 0.579 | -1.08 ± 6.6 | 0.579 |
| Huang et al. 2022 | HBP - 9 months | 32.6 ± 4.6 | 53.9 ± 11.9 | N/A | 21.3 ± 12.8 | BVP – 9 months | 34.6 ± 4.6 | 51.3 ± 7.4 | 0.015 | 16.7 ± 8.7 |
|  | HBP - 18 month | 32.6 ± 4.6 | 54.8 ± 9.4 | N/A | 22.2 ± 10.5 | BVP – 18 months | 34.6 ± 4.6 | 51.5 ± 14.7 |  | 16.9 ± 15.4 |
| Jin et al. 2022 | LBBAP | 53.0 ± 11.0 | 60.4 ± 8.8 | <0.001 | 7.4 ± 14.1 | N/A | N/A | N/A | N/A | N/A |
| Vijayaraman et al. 2022 | CSP - Overall | 46.5 ± 14.2 | 51.9 ± 11.2 | <0.05 | 5.4 ± 18.1 | CP – Overall | 36.4 ± 16.1 | 39.5 ± 16.0 | <0.05 | 3.1 ± 22.7 |
|  | HBP | 46.4 ± 14.1 | 52.3 ± 10.9 | <0.05 | 5.9 ± 17.8 | BVP | 26.7 ± 10.5 | 33.8 ± 15.5 | <0.05 | 7.1 ± 18.7 |
|  | LBBAP | 46.9 ± 15.1 | 50.1 ± 12.9 | <0.05 | 3.2 ± 19.9 | RVP | 50.3 ± 12.0 | 47.7 ± 13.2 | >0.05 | -2.60 ± 17.8 |
| Chaumont et al. 2023 | HBP - Overall | 47.0 ± 14.0 | 60.0 ± 9.0 | <0.001 | 13 ± 16.6 | N/A | N/A | N/A | N/A | N/A |
|  | HBP - Reduced LVEF | 27.0 ± 8.00 | 52.0 ± 7.00 | <0.001 | 25 ± 10.6 | N/A | N/A | N/A | N/A | N/A |
| Morina-Vazquez et al. 2021 | HBP - Overall | 60 (58-63.25) | 60 (60-65) | >0.05 | 0 | N/A | N/A | N/A | N/A | N/A |
|  | HBP - Reduced LVEF | 35 (23.8-45.3) | 40 (35-56.5) | <0.05 | 5 | N/A | N/A | N/A | N/A | N/A |
| Wu et al. 2021 | CSP - Overall | 34.3 ± 7.7 | 50.6 | <0.001 | 16.3 ± 7.7 | N/A | N/A | N/A | N/A | N/A |
|  | HBP | 35.4 ± 7.9 | 51.1 ± 12.2 | <0.001 | 15.7 ± 14.5 | N/A | N/A | N/A | N/A | N/A |
|  | LBBAP | 33.2 ± 7.2 | 49.7 ± 13.9 | <0.001 | 16.5 ± 15.7 | N/A | N/A | N/A | N/A | N/A |
| Su et al. 2020 | HBP - 1 year - Preserved LVEF | 56.6 + 10.3 | 62.3 ± 7.1 | <0.01 | 5.7 ± 12.5 | N/A | N/A | N/A | N/A | N/A |
|  | HBP - 2 year - Preserved LVEF | 56.0 ± 10.7 | 63.7± 8.1 | <0.01 | 7.7 ± 13.4 | N/A | N/A | N/A | N/A | N/A |
|  | HBP - 3 year - Preserved LVEF | 56.6 ± 10.5 | 63.3 ± 5.1 | <0.05 | 6.7 ± 11.7 | N/A | N/A | N/A | N/A | N/A |
|  | HBP > 3 year - Preserved LVEF | 57.1 ± 11.2 | 64.8 ± 6.7 | <0.05 | 7.7 ± 13.1 | N/A | N/A | N/A | N/A | N/A |
|  | HBP - 1 year - Reduced LVEF | 32.1 ± 6.10 | 49.1 ± 12.9 | <0.01 | 17 ± 14.3 | N/A | N/A | N/A | N/A | N/A |
|  | HBP - 2 year - Reduced LVEF | 32.6 ± 5.7 | 49.8 ± 12.2 | <0.01 | 17.2 ± 13.5 | N/A | N/A | N/A | N/A | N/A |
|  | HBP - 3 year - Reduced LVEF | 30.0 ± 5.80 | 49.9 ± 12.6 | <0.01 | 19.9 ± 13.9 | N/A | N/A | N/A | N/A | N/A |
|  | HBP > 3 year - Reduced LVEF | 29.5 ± 5.9 | 52.8 ± 14.2 | <0.01 | 23.3 ± 15.4 | N/A | N/A | N/A | N/A | N/A |
| Sun et al. 2020 | HBP - Overall | 48.8 ± 11.2 | 51.1 ± 9.0 | <0.01 | 2.3 ± 14.4 | N/A | N/A | N/A | N/A | N/A |
|  | HBP - Reduced LVEF | 34.4 ± 10.5 | 40.0 ± 10.0 | <0.01 | 5.6 ± 14.5 | N/A | N/A | N/A | N/A | N/A |
| Wang et al. 2019 | CSP - Overall | 34.8 ± 11.2 | 49.3 ± 14.9 | <0.001 | 14.5 ± 18.6 | N/A | N/A | N/A | N/A | N/A |
|  | CSP - Reduced LVEF | 29.8 ± 5.87 | 46.2 ± 14.7 | <0.001 | 16.4 ± 15.8 | N/A | N/A | N/A | N/A | N/A |
| Deshmukh et al. 2020 | HBP (+LV pacing) | 27.6 ± 6.4 | 42.6 ± 11.8 | <0.001 | 15 ± 13.4 | N/A | N/A | N/A | N/A | N/A |
| Huang et al. 2017 | HBP - Overall - 3 months | 44.9±14.6 | 56.5±8.7 | <0.001 | 11.6 ± 17 | N/A | N/A | N/A | N/A | N/A |
|  | HBP - Overall - 1 year | 44.9±14.6 | 59.7±9.8 | <0.001 | 14.8 ± 17.6 | N/A | N/A | N/A | N/A | N/A |
|  | HBP - Overall - Last F/U | 44.9±14.6 | 60.0±8.1 | <0.001 | 15.1 ± 16.7 | N/A | N/A | N/A | N/A | N/A |
|  | HBP - Preserved LVEF - 3 months | 56.6±9.9 | 60.1±8.0 | 0.231 | 3.5 ± 12.7 | N/A | N/A | N/A | N/A | N/A |
|  | HBP - Preserved LVEF - 1 year | 56.6±9.9 | 63.2±8.2 | 0.01 | 6.6 ± 12.9 | N/A | N/A | N/A | N/A | N/A |
|  | HBP- Preserved LVEF - Last F/U | 56.6±9.9 | 62.6±6.9 | 0.019 | 6 ± 12.1 | N/A | N/A | N/A | N/A | N/A |
|  | HBP - Reduced LVEF - 3 months | 32.2±4.8 | 53.9±8.4 | <0.001 | 21.7 ± 9.7 | N/A | N/A | N/A | N/A | N/A |
|  | HBP - Reduced LVEF - 1 year | 32.2±4.8 | 55.7±10.2 | <0.001 | 23.5 ± 11.3 | N/A | N/A | N/A | N/A | N/A |
|  | HBP - Reduced LVEF - Last F/U | 32.2±4.8 | 57.2±8.7 | <0.001 | 25 ± 9.9 | N/A | N/A | N/A | N/A | N/A |
| Vijayaraman et al. 2017 | HBP - Overall | 43.0 ± 13.0 | 50.0 ± 10.0 | 0.01 | 7 ± 16.4 | N/A | N/A | N/A | N/A | N/A |
|  | HBP - Preserved LVEF | 33.0 ± 7.00 | 57.0 ± 7.00 | 0.5 | 1 ± 9.9 | N/A | N/A | N/A | N/A | N/A |
|  | HBP - Reduced LVEF | 56.0 ± 5.00 | 45.0 ± 9.00 | <0.001 | 12 ± 10.3 | N/A | N/A | N/A | N/A | N/A |
| Occhetta et al. 2006 | HBP | 52.0 ± 9.1 | 53.4 ± 7.9 | >0.05 | 1.4 ± 12.1 | RVP | 52.0 ± 9.1 | 50.0 ± 7.9 | >0.05 | -2 ± 12.1 |
| LVEF *Left ventricular ejection fraction;* CSP *Conduction system pacing;* N/A *Not applicable/available;* BVP *Biventricular pacing; SR Sinus rhythm;* HBP *His Bundle pacing;* LBBAP *Left bundle branch associated pacing;* LV *Left ventricular;* F/U *Follow-up* | | | | | | | | | | |

| **Supplementary Table 5. Echocardiographic outcomes – Left ventricular dimensions** | | | | | | | | | | | | | | | | |  |
| --- | --- | --- | --- | --- | --- | --- | --- | --- | --- | --- | --- | --- | --- | --- | --- | --- | --- |
| **Study** | **Conduction System Pacing – LV Volume** | | | | | | | **Biventricular Pacing – LV Volume** | | | | | | | |  |  |
|  | **Specified intervention** | **Baseline LVEDV** | **F/U LVEDV** | **P values** | **Baseline LVESV** | **F/U LVESV** | **P values** | | **Specified intervention** | **Baseline LVEDV** | **F/U LVEDV** | **P values** | **Baseline LVESV** | **F/U LVESV** | **P values** | | |
| Palmisano, Parlavecchio, Crea et al. 2023 | CSP (HBP or LBBAP) | N/A | N/A | N/A | N/A | N/A | N/A | | N/A | N/A | N/A | N/A | N/A | N/A | N/A | | |
| Ivanovski et al. 2023 | CSP (HBP or LBBAP) | * SR group - 56 (51-69) NSR group - 76 (53-94) | *SR group - 53 (49-57) NSR group - 63 (47-82) | SR group -p=0.144 NSR group - p<0.001 | **SR group - 32 (27-41) NSR group - 47 (27-63) | **SR group - 25 (25-26) NSR group - 29 (21-46) | SR group -p=0.144 NSR group - p<0.001 | | N/A | N/A | N/A | N/A | N/A | N/A | N/A | | |
| Liu et al. 2023 | LBBAP | N/A | N/A | N/A | N/A | N/A | N/A | | N/A | N/A | N/A | N/A | N/A | N/A | N/A | | |
| Palmisano, Parlaveccio, Vetta et al. 2023 | CSP (HBP or LBBAP) | N/A | N/A | N/A | N/A | N/A | N/A | | N/A | N/A | N/A | N/A | N/A | N/A | N/A | | |
| Palmisano, Ziacchi et al. 2023 | CSP (HBP or LBBAP) | N/A | N/A | N/A | N/A | N/A | N/A | | N/A | N/A | N/A | N/A | N/A | N/A | N/A | | |
| Rijks et al. 2023 | LBBAP | N/A | N/A | N/A | N/A | N/A | N/A | | N/A | N/A | N/A | N/A | N/A | N/A | N/A | | |
| Qi et al. 2023 | CSP (HBP or LBBAP) | ***46.9 +/- 5.4 | ***46.0 +/- 4.0 | 0.22 | N/A | N/A | N/A | | N/A | N/A | N/A | N/A | N/A | N/A | N/A | | |
| Nam et al. 2023 | HBP with dsLBBAP | N/A | N/A | N/A | N/A | N/A | N/A | | N/A | N/A | N/A | N/A | N/A | N/A | N/A | | |
| Yang et al. 2023 | HBP + LBBAP | N/A | N/A | N/A | 137 ± 36.2 | HBP – 123 ± 21.8  LBBAP – 125 ± 26.0 | HBP – P=0.02  LBBAP- P=0.04 | | N/A | N/A | N/A | N/A | N/A | N/A | N/A | | |
| Cai et al. 2023 | HBP | N/A | N/A | N/A | N/A | N/A | N/A | | N/A | N/A | N/A | N/A | N/A | N/A | N/A | | |
|  | LBBAP | N/A | N/A | N/A | N/A | N/A | N/A | | N/A | N/A | N/A | N/A | N/A | N/A | N/A | | |
| Pillai et al. 2023 | CSP (HBP or LBBAP) | ***46 ± 6 | ***44 ± 7 | 0.25 | ****32 ± 6 | ****32 ± 9 | 0.93 | | N/A | N/A | N/A | N/A | N/A | N/A | N/A | | |
| Ivanovski et al. 2022 | HBP | *72 ± 21 | *61 ± 18 | 0.006 | **45 ± 18 | *32 ± 13 | <0.001 | | CRT | *82 ± 17 | *84 ± 19 | 0.509 | **51 ± 12 | **53 ± 14 | 0.551 | | |
|  | LBBAP | *89 ± 22 | *81 ± 21 | 0.002 | **62 ± 21 | *50 ± 18 | 0.004 | |  |  |  |  |  |  |  |  |  |
| Zizek et al. 2022 | HBP | *75 (54.7-94.3) | *63.6 (49.6-81) | 0.013 | **45.5 (33-55.3) | **32.7 (25.6-42.6) | 0.005 | | CRT | 75.9 (65.3 -93) | 79.9 (66-100) | 0.225 | 47.9 (39.5 - 55.7) | 46.4 (42.9 - 68.1) | 0.281 | | |
| Huang et al. 2022 | HBP | ***62.5 ± 8.5 | ***54.0 ± 5.90 | <0.001 | N/A | N/A | N/A | | CRT | ***61.5 ± 7.50 | ***55.7 ± 2.60 | <0.001 | N/A | N/A | N/A | | |
| Jin et al. 2022 | LBBAP | ***49.5 ± 5.80 | ***46.3 ± 5.60 | <0.001 | N/A | N/A | N/A | | N/A | N/A | N/A | N/A | N/A | N/A | N/A | | |
| Vijayaraman et al. 2022 | CSP (HBP or LBBAP) | N/A | N/A | N/A | N/A | N/A | N/A | | N/A | N/A | N/A | N/A | N/A | N/A | N/A | | |
| Chaumont et al. 2022 | HBP | N/A | N/A | N/A | N/A | N/A | N/A | | N/A | N/A | N/A | N/A | N/A | N/A | N/A | | |
| Morina-Vazquez et al. 2021 | HBP | N/A | N/A | N/A | N/A | N/A | N/A | | N/A | N/A | N/A | N/A | N/A | N/A | N/A | | |
| Wu et al. 2021 | CSP (HBP or LBBAP) | Narrow complex 152.5 ± 50.6  LBBB 173.9 ± 70.8 | Narrow complex 129.1 ± 53.2  LBBB 131.2 ± 54.1 | 0.028 | Narrow complex 99.7 ± 40.8  LBBB 113 ± 55.7 | Narrow complex 72.5 ± 48.6  LBBB 61.7 ± 33.2 | 0.002 | | N/A | N/A | N/A | N/A | N/A | N/A | N/A | | |
| Su et al. 2020 | HBP | ***55.8 ± 9.50 | ***51.8 ± 8.50 | 0.0008 | N/A | N/A | N/A | | N/A | N/A | N/A | N/A | N/A | N/A | N/A | | |
| Sun et al. 2020 | HBP | N/A | N/A | N/A | N/A | N/A | N/A | | N/A | N/A | N/A | N/A | N/A | N/A | N/A | | |
| Wang et al. 2019 | HBP | N/A | N/A | N/A | 122.7 ± 65.2 | 83.7 ± 62.5 | <0.001 | | N/A | N/A | N/A | N/A | N/A | N/A | N/A | | |
| Deshmukh et al. 2020 | HBP | 172 ± 86.1 | 136 ± 69.7 | <0.05 | N/A | N/A | N/A | | N/A | N/A | N/A | N/A | N/A | N/A | N/A | | |
| Huang et al. 2017 | HBP | ***55.8 ± 8.10 | ***51.0 ± 5.10 | <0.001 | N/A | N/A | N/A | | N/A | N/A | N/A | N/A | N/A | N/A | N/A | | |
| Vijayaraman et al. 2017 | HBP | N/A | N/A | N/A | N/A | N/A | N/A | | N/A | N/A | N/A | N/A | N/A | N/A | N/A | | |
| LVEF *Left ventricular ejection fraction;* CSP *Conduction system pacing;* N/A *Not applicable/available;* BVP *Biventricular pacing; SR Sinus rhythm;* HBP *His Bundle pacing;* LBBAP *Left bundle branch associated pacing;* LV *Left ventricular;* F/U *Follow-up; **LVEDVi (left ventricle end diastolic volume index) (mL/m2); **LVESVi (left ventricle end systolic volume index) (mL/m2); ***LVEDD (left ventricle end diastolic diameter); **** LVESD (left ventricular end systolic diameter) | | | | | | | | | | | | | | | | |  |

| **Supplementary Table 6.** The significance of potential sources of heterogeneity tested by meta-regression | | | |
| --- | --- | --- | --- |
|  | **Change in LVEF** | **Change in QRSd** | **Change in NYHA** |
| **Test Factor** | **P value** | | |
| Mean Age | 0.070 | 0.309 | **0.009** |
| Male proportion | 0.687 | 0.391 | **0.013** |
| Baseline LVEF | 0.220 | 0.520 | 0.412 |
| Baseline QRSd | **0.0490** | **0.0470** | 0.934 |
| LVEF *Left ventricular ejection fraction;* QRSd *QRS duration;* NYHA *New York Heart Association class* | | | |
